# Large scale *in silico* characterization of repeat expansion variation in human genomes

**DOI:** 10.1038/s41597-020-00633-9

**Published:** 2020-09-08

**Authors:** Sarah Fazal, Matt C. Danzi, Vivian P. Cintra, Dana M. Bis-Brewer, Egor Dolzhenko, Michael A. Eberle, Stephan Zuchner

**Affiliations:** 1 Dr. John T. Macdonald Foundation Department of Human Genetics and John P. Hussman Institute for Human Genomics, University of Miami Miller School of Medicine, Miami, FL USA; 2 Illumina Inc., San Diego, CA USA

**Keywords:** Structural variation, Microsatellite instability

## Abstract

Significant progress has been made in elucidating single nucleotide polymorphism diversity in the human population. However, the majority of the variation space in the genome is structural and remains partially elusive. One form of structural variation is tandem repeats (TRs). Expansion of TRs are responsible for over 40 diseases, but we hypothesize these represent only a fraction of the pathogenic repeat expansions that exist. Here we characterize long or expanded TR variation in 1,115 human genomes as well as a replication cohort of 2,504 genomes, identified using ExpansionHunter Denovo. We found that individual genomes typically harbor several rare, large TRs, generally in non-coding regions of the genome. We noticed that these large TRs are enriched in their proximity to *Alu* elements. The vast majority of these large TRs seem to be expansions of smaller TRs that are already present in the reference genome. We are providing this TR profile as a resource for comparison to undiagnosed rare disease genomes in order to detect novel disease-causing repeat expansions.

## Introduction

Variation in protein coding exons accounts for the majority of the known pathogenic changes causing rare monogenic disorders. As approximately 50% of the 7,000 clinically defined diseases remain genetically unexplained^1^, we and others have speculated that non-coding sequence variation is contributing more significantly to the pathogenic variant spectrum^2,3^. Structural variation is already shown to play an important role in non-coding regions of the genome^4^. One form of structural variation is the expansion of short tandem repeats (TRs) into long repetitive stretches of the wild-type consensus sequence or a mutated version of the motif. Over 40 known diseases are associated with repeat expansions, most of which primarily affect the nervous system^5^. Prominent examples include Friedreich ataxia (FRDA) caused by a GAA expansion in intron 1 of the *FXN* gene^6^, amyotrophic lateral sclerosis (ALS) caused by a GGGGCC expansion in intron 1 of the *C9ORF72* gene^7^, and myotonic dystrophy type 2 (DM2) caused by a CCTG expansion in intron 1 of the *ZFN9* gene^8^. We speculate that there are more repeat expansion loci yet to be discovered because there are over 500,000 TRs mapped in the human genome and these regions are highly mutable^9^.

Allelic heterogeneity in TRs is far greater than the variation seen in typically biallelic single nucleotide polymorphisms (SNPs), making them historically valuable markers for use in linkage studies and forensics. There is large variability in TR lengths because the mutation rates affecting TRs is 10 to 10,000-fold higher than the mutation rates of nonrepetitive regions^10^. Two major mechanisms are responsible for increasing the variability in repeat sequences: slipped strand mispairing (SSM) and homologous recombination (HR). SSM is the process by which TRs contract and expand during replication due to loop formation of the repetitive sequence. Depending on whether the loop is formed on the template or daughter strand, the TR will consequently undergo a contraction or expansion respectively. HR occurs as a repair mechanism after double strand breaks in the DNA^11^. It can introduce repeat length alterations through misalignment during the strand invasion phase^12^. Factors that influence the instability of TRs include number of repeats in the sequence, motif length, and purity of the repeat sequence. Longer repeats with high purity are far more unstable than shorter repeats with lower purity^10^.

To date, repeat expansion loci have been discovered mostly by deeply evaluating linkage loci in families. Direct identification from short-read whole genome sequence data has proven difficult due to the inefficiency of alignment algorithms to correctly match repeat-rich reads to the reference genome. However, with the advance of new tools such as ExpansionHunter some of these challenges can be overcome^13^. Long read sequencing technology such as those offered by PacBio and Oxford Nanopore are also able to circumvent these obstacles, but they are still costly at the human genome level and prone to errors^14^.

ExpansionHunter is a tool which predicts allele sizes at user-defined TR loci. It uses three primary types of evidence present in short read sequencing data, termed spanning reads, flanking reads, and in-repeat reads (IRRs). Spanning reads are those which contain the repeat sequence flanked on both ends by non-repetitive sequence. Flanking reads are those which contain repeat sequence on one end, and uniquely-mappable sequence on the other. IRRs are those which are composed almost entirely of the repetitive sequence. Since these analyses are performed using paired-end sequencing data, IRRs can sometimes be ‘anchored’ by their mate to a specific locus with high confidence^13^. ExpansionHunter Denovo (EHDn) is a variant of the ExpansionHunter method which exclusively utilizes the anchored IRRs to search the whole genome for evidence of large repetitive loci that are longer than the read length. It identifies such sites through clusters of anchor reads whose mates share a common repetitive motif and is able to assign approximate locations to each identified locus. In this way, EHDn is not constrained by read length or a pre-determined list of TR loci in its ability to identify expanded TRs^15^.

Here we have used ExpansionHunter Denovo to characterize long or expanded TR variation in 1,115 human genomes as well as a replication cohort of 2,504 genomes. Currently there is insufficient data on the diversity of repeat sequences in a control population. Although there are several published studies cataloging TR variation in controls, these are not comprehensive as they have mostly focused on short repeats detectable within spanning reads and repeats at predefined loci^16–21^. Our method on the other hand identifies repeats larger than the read length on a genome-wide scale. We propose to use this TR profile as a comparative control dataset in the search for new pathogenic repeat expansions in undiagnosed rare Mendelian diseases, in particular neurodegenerative disorders.

## Results

### Identification of large TRs

We applied EHDn to a set of 1,115 WGS samples, searching genome-wide for evidence of large, repetitive sites with a motif length of 3–8 base pairs. Our goal was to characterize the landscape of large TRs detectable with EHDn in these samples, for use as a reference in future studies. To assess the reproducibility of our results, we additionally applied EHDn to the deeply sequenced set of 2,504 WGS samples from Phase 3 of the 1000 Genomes Project. In our dataset of 1,115 individuals, we identified a total of 4,604 large TRs that passed our filtering criteria (Supplementary Table 1^22^, Supplementary File 1). In the 1000 Genomes sample set, we identified a total of 2,436 large TRs that passed our filtering criteria (Supplementary Table 2^22^, Supplementary File 2). We selected for TRs spanning more than approximately 175 bp in at least one sample by requiring a minimum of five anchored IRRs supporting the expansion event (see Methods). Given that our data utilizes 150 bp paired-end read data sequenced to an average depth of 40x (Table 1), we believe that these criteria would safely eliminate any loci where the repetitive sequence is shorter than the read length. Evidence supporting this assertion is described in detail by Dolzhenko *et al*. in their publication introducing ExpansionHunter Denovo^15^.

**Table 1:** Descriptions of the sample sets used.

Sample Set	Number of Samples	Sequencing Platform	PCR-free	Read Length	Goal Coverage	Average Observed Coverage
Vanderbilt Atrial Fibrillation Registry	1,115	HiSeq X Ten	Yes	150 bp paired	~30X	40X
1000 Genomes Project Phase 3	2,504	NovaSeq	Yes	150 bp paired	~30X	38X
1000 Genomes long read data	5	PacBio Sequel2 HiFi	Yes	Various (~10 kb)	~30X	30–45X

For each sample set, the number of samples, sequencing platform, PCR use in library production, read length, and intended and observed sequencing coverage are given.

As an additional test of whether the presence of at least five anchored IRRs indicates the existence of a repetitive region of at least 175 bp, we utilized PacBio HiFi high coverage, long read data on five of the samples from the 1000 Genomes cohort. We checked the allele sizes of the 15 rare TRs called by EHDn as well as 35 randomly selected common TRs called by EHDn (supported by at least 5 anchored IRRs) in these samples, for a total of 50 sites inspected. We found that 14 out of the 15 rare sites and 34 out of the 35 common sites had at least one allele greater than 175 bp in the PacBio data, in line with our expectation. The rare site and common site that did not have an allele greater than 175 bp each had two alleles of approximately 170 bp and 163 bp respectively. This demonstrates how smaller homozygous repetitive regions can appear similar to larger heterozygous ones when using EHDn. Even so, all inspected sites showed an expansion of at least 160 bp for at least one allele in the PacBio data indicating that even if our minimum size expectation of 175 bp is a bit inflated, the software is still detecting a repetitive region longer than the read length. The EHDn results and PacBio allele sizes for the inspected sites are reported in Supplementary Table 3. This analysis suggests that the database of repetitive regions identified here is likely to have high precision.

### Individual genomes typically harbor several rare, large TRs

**Fig. 1:**
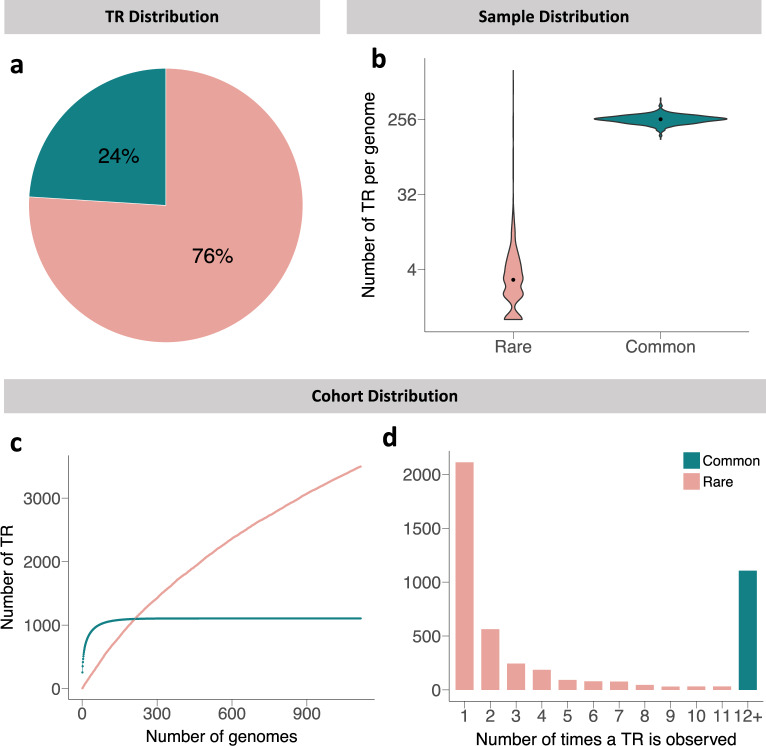
Distributions of rare and common TRs. (**a**) Percentage distribution of TRs into the rare and common subcategories. (**b**) Number of TRs per genome in each category. (**c**) Number of TRs as a function of sample size. (**d**) Frequency plot of the number of times a TR is observed in the cohort.

Our first goal in the analysis of this data was to characterize the occurrence rates and locations of large, repetitive loci in a typical genome. We divided the 4,604 TRs in our dataset into two categories; rare and common. Here, we defined rare and common as occurring in less or more than 1% of the genomes, respectively. Figure 1 shows the distributions of rare and common TRs within each genome and among the cohort as a whole. We found 1,106 common TRs and 3,498 rare TRs (Fig. 1a). Each genome contains a median of 3 rare TRs and 256 common TRs (Fig. 1b). The narrow distribution of common TRs (147–460) indicates that their frequency is surprisingly evenly represented among all individuals. Conversely, although most genomes contain a small number of rare TRs, we observed a subset of outliers with much higher counts (0–986). Analysis of 2,504 WGS samples from the 1000 Genomes Project gives us a similar distribution with the exception of these outliers (Supplementary Figure 1a,b). Both datasets have a median of 3 and an interquartile range of 3 for rare TRs. The full range for the 1000 Genomes Project dataset however is 0–14, which is much more tightly distributed than our 1,115 WGS dataset. This discrepancy is most likely due to more intensive QC filtering for the 1000 Genomes Project. There is high overlap of the common sites observed in both datasets, with 80% of the sites identified as common in the 1000 Genomes Project also existing in the 1,115 WGS dataset (data not shown).

As we look at TR variation in the entire dataset of 1,115 genomes, we can estimate the number of samples that would contain most of the TRs in each subset. Figure 1c shows the number of large TRs discovered as a function of sample size. We find that the identification of common large TRs is quickly saturated. Exploration of about 450 genomes identifies all common large TRs in our sample. Conversely, novel rare TRs continue to be identified at a rate of about 1.8 per additional genome after inspection of all 1,115 samples here. We also examined how frequently rare TRs occurred in the entire sample and found that about half of them are uniquely represented in a single genome (Fig. 1d). This frequency decreases in a stepwise pattern as the number of occurrences increases up to 12, at which point the TRs are considered commonly represented. These patterns are also reproduced in the 1000 Genomes Project data (Supplementary Figure 1c,d).

### Most identified large TRs are located in non-coding regions of the genome

**Fig. 2:**
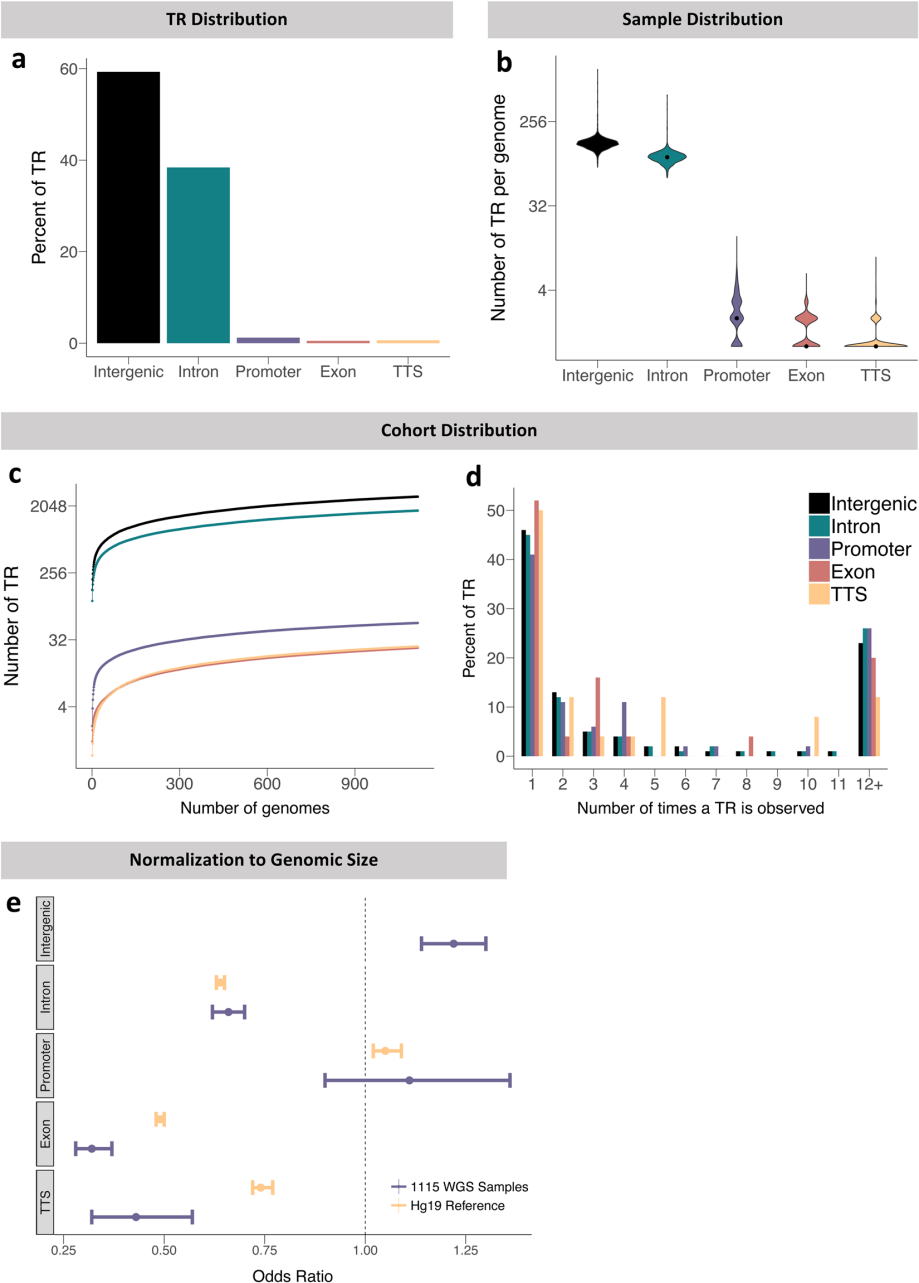
Distributions of TRs in different genomic regions; intergenic, intron, promoter, exon, and TTS. (**a**) Percentage distribution of TRs into the genomic region subcategories. (**b**) Number of TRs per genome in each category. (**c**) Number of TRs as a function of sample size. (**d**) Frequency plot of the number of times a TR is observed in the cohort. (**e**) Odds ratios calculated by Fisher’s exact test for TRs in different genomic regions, in both our dataset and the hg19 reference genome. There is no odds ratio produced for the intergenic category of hg19 because the number of overlaps between the simple repeats and intergenic regions exceeds that of the total number of intergenic regions. This produces a contingency table that results in an undefined value for the odds ratio.

Next, we categorized the TRs into the regions of the genome in which they are located and completed similar analyses as done for Fig. 1 to generate a comprehensive view of the sample and cohort distributions. We found that 97.7% of our large TRs are located in intergenic and intronic regions (Fig. 2a). This is approximately in agreement with other sources which have found that only about 8% of total TRs in the genome are located in coding regions^23^. Some discrepancy is expected since we are only examining TRs above ~150 bp in length. When we inspected the locations of these expanded loci, we found that intergenic and intronic TRs are most abundantly represented in each genome at a median of 148 and 106, whilst large TRs in the promoter, exon, and transcription termination site (TTS) are a minority at a median of 2, 1, and 1, respectively (Fig. 2b). After exploration of 1,115 genomes, the number of TRs does not reach saturation and 0.01–1 novel TRs are expected with each additional genome to each genomic region analyzed. The majority of the unseen TRs are expected in intergenic and intronic regions, while exons expect the fewest (Fig. 2c). Analysis of the number of occurrences per TR demonstrated the same decreasing stepwise pattern as seen in the rare vs common comparison, with 40–50% of TRs being represented as singletons. In each categorical subgroup, we see a similar distribution of about 74–88% of TR occurring in 11 or fewer genomes (less than 1% of the total sample size). These observations largely replicated in the 1000 Genomes dataset (Supplementary Figure 2a–d), with the exception that TRs overlapping TTS elements were mostly observed in two individuals instead of as singletons in the original sample set.

Due to the differences in the proportion of the genome each genomic region comprises, absolute numbers of TRs observed in each group is expected to greatly vary. We used Fisher’s exact test to adjust for genomic space and calculate the odds ratios for the TRs detected in the different genomic regions (Fig. 2e). This standardizes the comparison between the categorical subsets which differ dramatically in the fraction of the genome they cover. We discovered that TRs in exons and TTSs were found less frequently than expected by random chance with odds ratios of 0.32 and 0.43, respectively. This underrepresentation is not surprising as coding sequences are more highly conserved and typically do not harbor longer TRs because expansions may infer pathogenicity^24,25^. This pattern of underrepresentation of large TRs in exonic and TTS regions also replicated in the 1000 Genomes dataset (Supplementary Figure 2e).

### There is an enrichment for TRs overlapping *Alu* elements

**Fig. 3:**
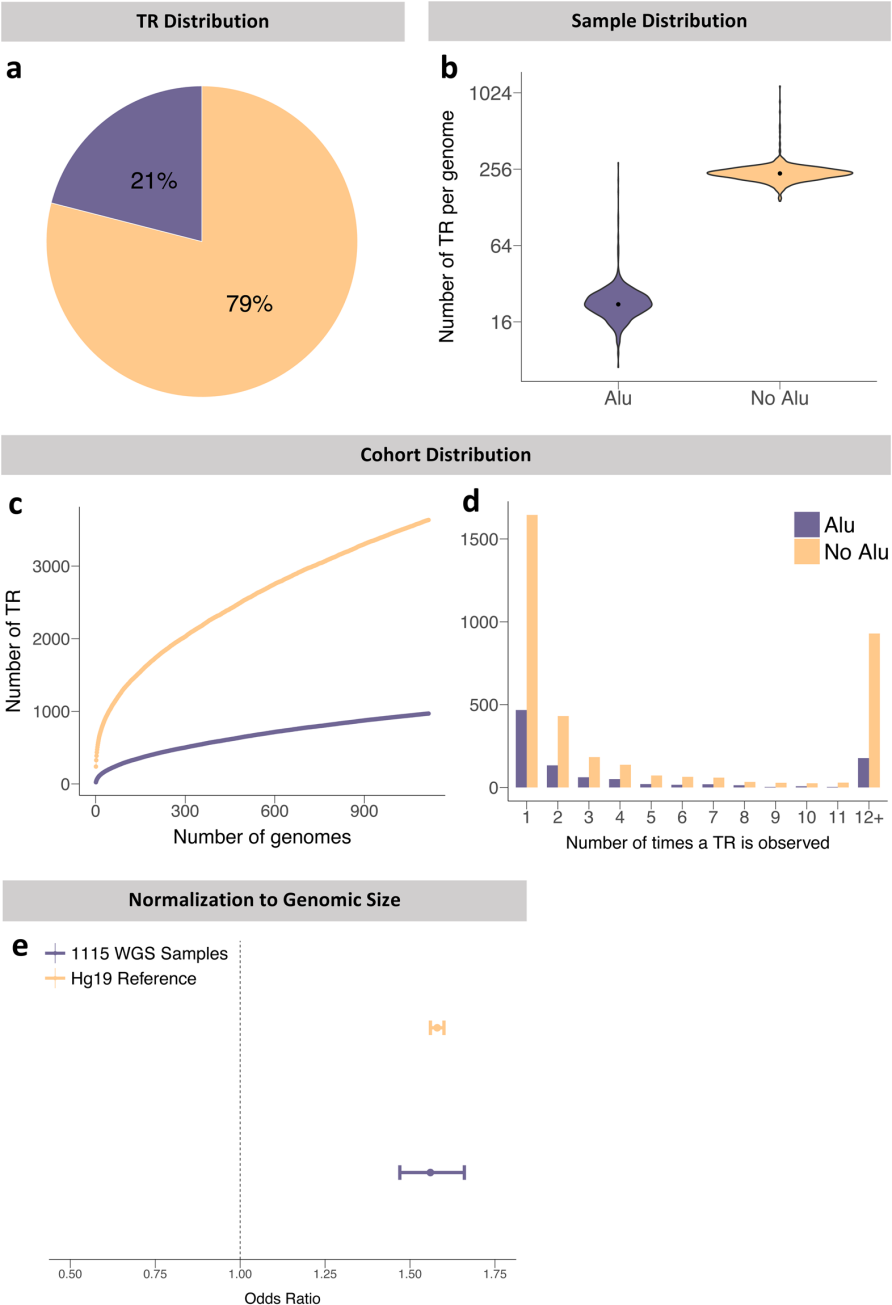
Distributions of TRs in *Alu* and non-*Alu* overlapping regions. (**a**) Percentage distribution of TRs into the subcategories. (**b**) Number of TRs per genome in each category. (**c**) Number of TRs as a function of sample size. (**d**) Frequency plot of the number of times a TR is observed in the cohort. (**e**) Odds ratios calculated by Fisher’s exact test for TRs in each category, in both our dataset and the reference genome.

Finally, we decided to interrogate the localization of each large TR in relation to a neighboring *Alu* element because of their suspected role in the TR expansion process. *Alus* are long repetitive sequences that have the capability of jumping from one part of the genome to another^26^. There are currently 13 known diseases that are caused by repeat expansions in intronic sequences. Nine of them either overlap or border an *Alu* element, suggesting a connection between *Alu* elements and repeat expansion disorders (Supplementary Table 4)^21,27,28^. Upon assorting the TRs, 969 of the 4,604 total TRs were found to overlap an *Alu* element within ExpansionHunter Denovo’s relatively broad confidence interval for TR localization (Fig. 3a). We discovered that each genome in our dataset contained a median of 22 TRs which overlap *Alu* elements, and 237 TRs which did not (Fig. 3b). After exploring 1,115 genomes, neither group reached saturation, with 0.39 novel TRs expected to overlap with *Alus* per additional genome, and 1.34 non-*Alu* overlapping TRs expected (Fig. 2c). Analysis of the frequency of TRs occurring in the sample size again demonstrated the same decreasing stepwise pattern as seen for rare TRs, with approximately half occurring as singletons in each category (Fig. 3d). As with the normalization done for the genomic regions, we standardized the comparison between the two groups as *Alus* only comprise 11% of the genome and are therefore expected to have fewer absolute numbers of TRs^29^. We found an enrichment for TRs that overlap *Alus* in our dataset with an odds ratio of 1.56 (Fig. 3e). The patterns observed in the 1,115 genomes replicated in the 1000 Genomes dataset where we saw a similar proportion of large TRs overlapping *Alu* elements (21%), which again constituted an enrichment for *Alus* with an odds ratio of 1.90 (Supplementary Figure 3).

### Most identified large TRs are composed of two unique nucleotides, regardless of motif length

**Fig. 4:**
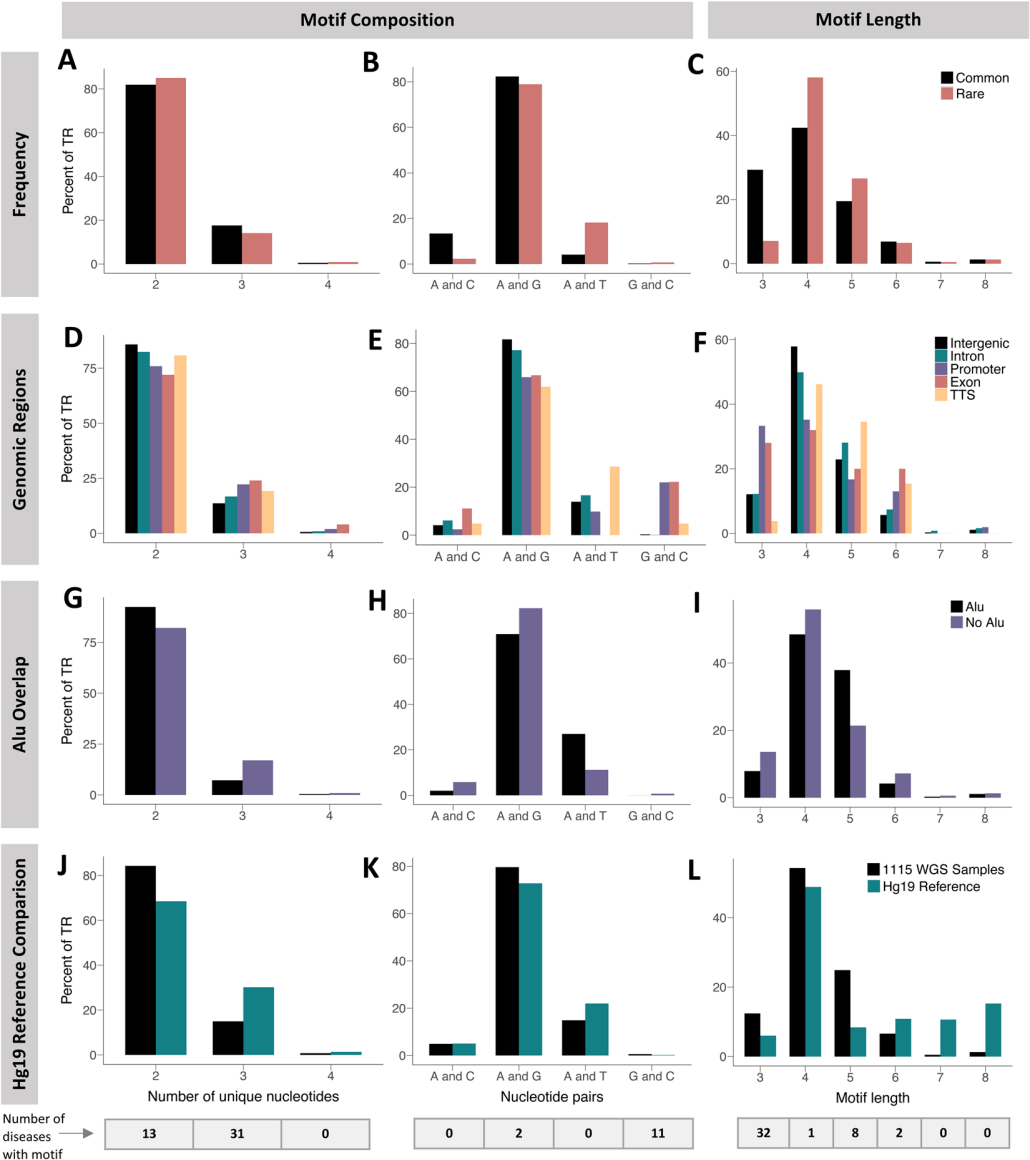
Characterization of observed motifs. Comparison of motifs (**A**–**C**) belonging to rare versus common TRs, (**D–F**) stratified by genomic region, (**G–I**) overlapping an *Alu* element or not, and (**J–L**) observed in the 1,115 WGS samples versus those in the hg19 reference genome. In each set of comparisons, the first column (**A,D,G**,**J**) shows the frequency of motifs composed of different numbers of unique nucleotides; the second column (**B,E,H**,**K**) presents the frequency of motifs of different pairs of nucleotides; and the third column (**C,F,I,L**) plots the frequency of motifs of lengths 3–8 bp. At the bottom, the number of known repeat expansion diseases with motifs fitting each subcategory described on the X axis is provided.

Next, we analyzed the motifs that were represented in our dataset of 4,604 TRs. We assessed the number of unique nucleotides that the motifs were composed of, as well as motif length. We find that in all subcategories of our comparative groups, over 70% of the motifs were composed of two unique nucleotides (Fig. 4A,D,G). We next investigated which pairs of nucleotides were represented. In all subcategories, the majority of motifs are composed of A and G pairs (Fig. 4B,E,H). Interestingly, promoter and exon regions contain over 20% of TR motifs with G and C pairs, in contrast to less than 1% for all other categorical groups (Fig. 4E). Of the four amino acids that are coded by purely GC containing triplets, three are typically enriched in repetitive regions of exons (arginine, alanine, and proline)^11,30^. When comparing our TR dataset as a whole to the TRs in the reference genome (from the UCSC hg19 SimpleRepeats database), we find that the two datasets are in agreement that over 80% of the TRs are composed of two unique nucleotides (Fig. 4J). There is also a consensus on the overall scarcity of GC pairs, which could be a protective mechanism of the genome, as we noted that eleven of the currently known repeat expansion diseases are caused by pathogenic expansion of pure GC containing repeats (Supplementary Table 4). Finally, we see that more TRs in our dataset are composed of A and G pairs in comparison to the reference TRs (Fig. 4K). Note that this discrepancy could be a result of differences in sequencing technology that was used to produce the two datasets.

### Most identified large TRs are composed of motifs that are 4 base pairs long

When looking at motif length, we find that the majority of TRs in our dataset are 4 nucleotides in length (Fig. 4C,F,I). Exons and promoters have a much higher representation of motifs of length 3 and 6 compared to intergenic and intronic regions, which indicates that the repeats in coding regions have a tendency towards being in frame (Fig. 4F). When comparing with the hg19 reference, we see general agreement that the majority of motifs are 4 nucleotides long, followed by motifs that are 5 nucleotides long. However, the hg19 reference contains more TRs with motifs that are 6, 7, and 8 base pairs long (Fig. 4L). This could be a result of either issues with the sequencing technology, alignment algorithms, or may be a discrepancy resulting from our method only focusing on detecting larger repetitive sequences.

### The majority of the large TRs stem from a reference repeat locus

**Fig. 5:**
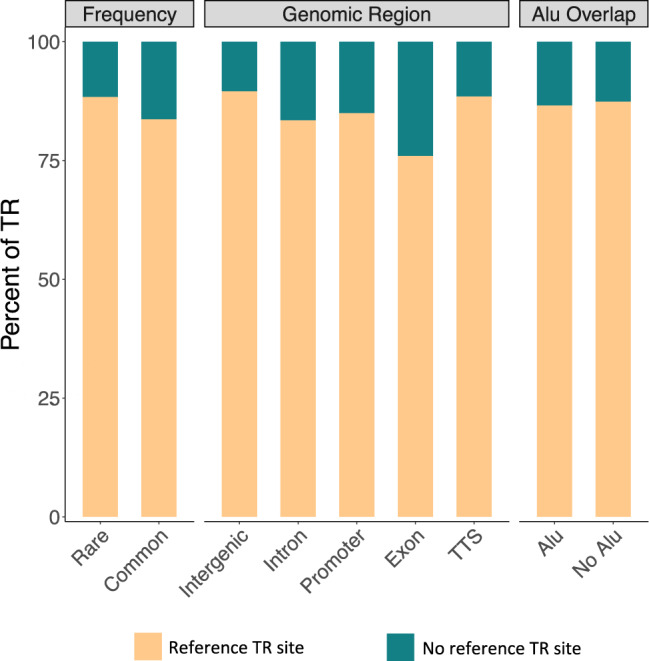
Percent of TRs originating from reference repeat loci with matching motifs in categorical subsets of frequency, genomic regions, and *Alu* overlap.

There are currently over 40 known diseases that are caused by repeat expansions^5^. These pathogenic repeats are present in the reference genome at shorter lengths. While most of the known diseases are caused by an expansion of the same motif as the reference, there are several which require the expansion motif to be mutated (Supplementary Table 4). One such example is a form of cerebellar ataxia with neuropathy and bilateral vestibular areflexia (CANVAS) syndrome, which is caused by an expansion and mutation of an AAAAG motif to AAGGG in intron 2 of the *RFC1* gene^19^. In other cases such as spinocerebellar ataxia 31 (SCA31), the pathogenic TGGAA repeat is an insertion flanked by the reference TAAAA repeat^31^. A major advantage of ExpansionHunter Denovo is its ability to detect repeats that do not match the reference locus, therefore enabling us to discover pathogenic repeats similar to those found in CANVAS and SCA31^15^. We were curious as to what proportion of our observed TRs originated from a reference locus. We compared them to simple repeats found in the hg19 reference genome and counted the number of overlapping sites which also shared the same motif or the reverse complement. We found that in all the subgroups, 76–90% of the TRs overlapped with a reference repeat locus (Fig. 5). The exonic TRs had the least overlap, which was contrary to our expectation as coding regions are more conserved than non-coding regions^32^. Additionally, TRs in intergenic regions had the highest percentage of overlap. It is important to note that the hg19 reference genome is composed of sequences from 13 individuals and is therefore not a comprehensive representation of the variation in the general population^33^.

### Detection of large TRs at known disease loci

**Fig. 6:**
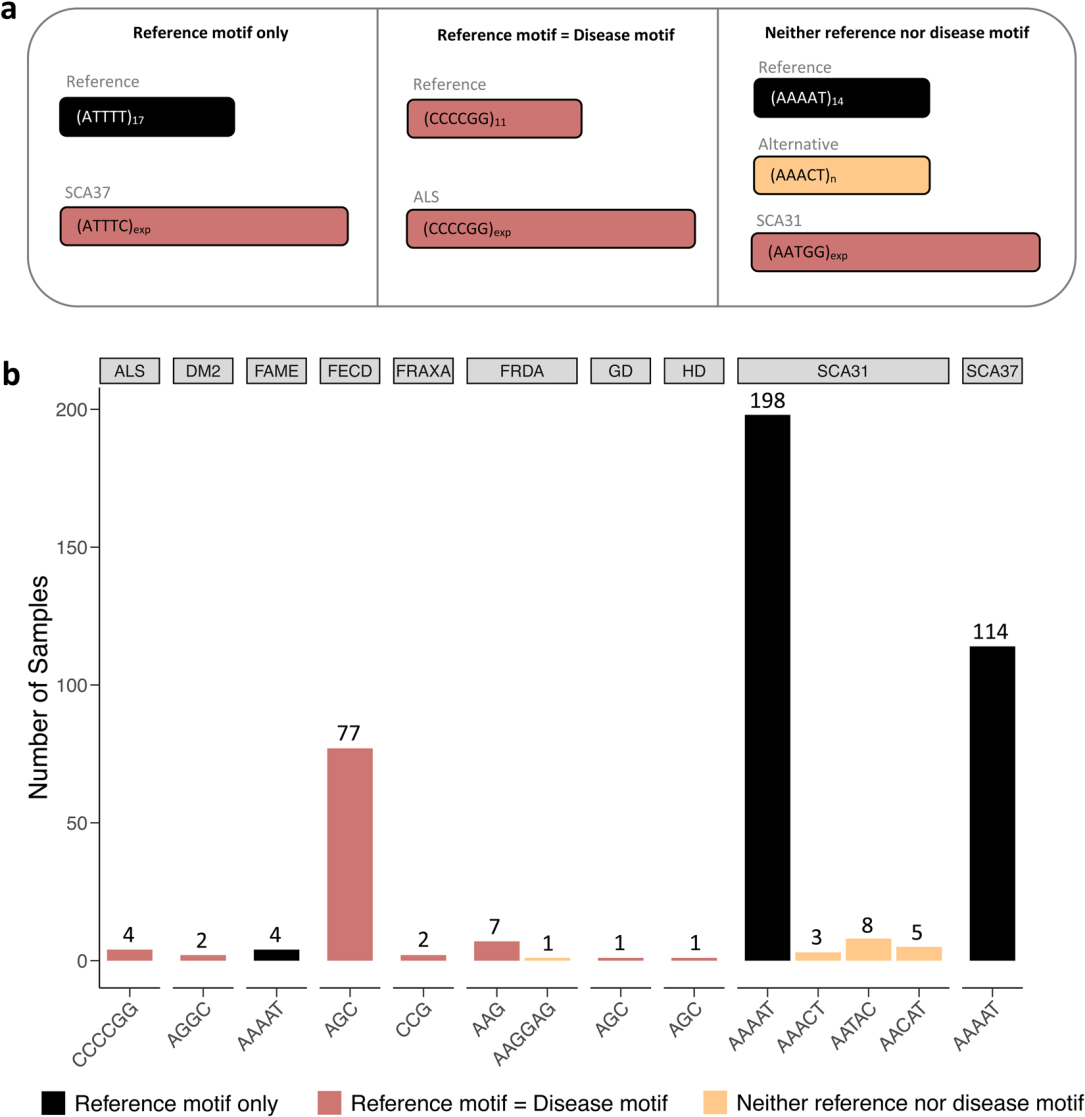
TR variation at known disease loci. (**a**) Schematic demonstrating the color-coded legend for panel B. *Reference motif only* refers to loci that require a mutated motif for disease manifestation. *Reference motif = Disease motif* refers to loci that require an expanded version of the control motif for disease manifestation. *Neither reference nor disease motif* refers to loci that have alternative motifs that differ from the reference and disease motifs but do not result in disease. (**b**) Frequency of genomes with TRs larger than the read length at known disease loci.

Next, we searched through our dataset of TRs for those which belonged to the locus of any known repeat expansion disease. We found 11 of these disease loci represented in our set of 4,604 TRs. Figure 6 shows ten of these disease loci and the number of genomes in our sample size which had evidence of more than 5 IRRs (data for CANVAS which is the final disease can be found in Supplementary Figure 5). Seven of these diseases are caused by an expansion of the same motif found in the reference at that locus (reference motif = disease motif). In each of these cases, there were a small number of samples which contained reads supporting evidence of a repeat size larger than previously reported for controls. In the case of glutaminase deficiency, one genome contained approximately 126 repeats which is far greater than the 8–16 reported for controls, but much smaller than the 680–1500 required to cause the disease^34^. For myotonic dystrophy 2 (DM2), where the standard range is <26 repeats we found two samples in our dataset who have up to 69 repeats, which is still below the 75 repeats required for pathogenicity^8^. At the five other disease loci, we see genomes with repeat sizes that have been reported as pathogenic. For example, at the Fuchs endothelial corneal dystrophy (FECD) locus, 6.9% of the 1115 WGS samples, and 3.5% of the 1000 Genomes samples have estimated repeat sizes in the pathogenic range. However, this is in alignment with the 3% allele frequency reported for controls in the literature^35^. FECD is a late-onset common disease and the expansion is therefore expected to be seen in our cohorts. At the Fredrich’s ataxia (FRDA) locus there are 3 genomes with reads supporting an expected size of 64–101 repeats, whilst the currently reported normal range of repeats is 6–27^36^. Although patients manifesting FRDA typically contain 200–900+ repeats, there have been reports of cases with as few as 44 repeats^36,37^. Note that several repeat expansion diseases, including FRDA, are autosomal recessive and ExpansionHunter Denovo cannot distinguish between sizes of the two alleles. The read counts that it returns typically correlate with the size of the larger allele (Supplementary Figure 4). As a result, follow up with other tools and ultimately PCR validation are necessary. We cannot conclusively suggest that these individuals are at risk because the results have not been validated by PCR as we do not have access to the sample DNA. Additionally, it is uncertain whether individuals with smaller expansions are guaranteed to manifest the disease, hence there is controversy regarding the cut off values. For example, in the case of amyotrophic lateral sclerosis (ALS), some sources suggest that 30 repeats are sufficient to cause disease whilst others choose a more conservative value of 60^38^. Finally, a small percentage of controls have been reported with expansions at the loci for ALS and Fragile X^39–41^. Therefore, it may be natural to find rare incidences of repeat expansions in healthy individuals at noncoding disease loci.

Further evidence of this hypothesis is given in Supplementary Figure 6a where we analyzed the genomes of 21 individuals with validated cases of FRDA with ExpansionHunter Denovo and compared the number of anchored IRRs seen in each sample with those detected in our 1,115 WGS and 1000 Genomes cohorts. We see that while there are several samples in each of our cohorts that have more than 5 anchored IRRs at the FRDA locus (with the disease motif), there is a clear separation between the median FRDA case and even the most extreme result seen in our two cohorts. Five or fewer samples from each cohort have more IRRs than the FRDA sample with the fewest observed IRRs. This shows that while some individuals show evidence of an expansion at a known disease locus, there is still a large difference between the signal observed for real cases and what is seen in these control cohorts.

We used ExpansionHunter version 3 to estimate the repeat sizes of both alleles in the samples which EHDn flagged as pathogenic (Supplementary Figure 6b). We found that the controls which have expansions at the FRDA locus are carriers and only have one allele in the pathogenic range. The sample that was flagged for Huntington disease was determined to be a false positive. Finally, the samples predicted to have pathogenic sizes at the ALS and fragile X loci by EHDn were confirmed by ExpansionHunter 3. Table 2 shows how many samples in each cohort have a previously known potentially pathogenic motif, along with an explanation of the typicality of each result.

**Table 2:** Presence of pathogenic TRs in the 1115 WGS and 1000 Genomes cohorts.

Disease	Disease name	Inheritance	Motif	1115 WGS Samples	1000 Genomes	Explanation
				Number of samples with reads above pathogenic range	Number of samples with reads above pathogenic range	
ALS	Amyotrophic lateral sclerosis	Autosomal dominant	CCCCGG	4	3	There are a small number of controls reported to have pathogenic sizes for this condition
FRAXA	Fragile X (site A)	X-linked dominant	CCG	2	2	These samples have repeats above the pathogenic cut-off for Fragile-X tremor ataxia syndrome. There are a small number of controls reported to have pathogenic sizes for Fragile-X
FRDA	Friedrich’s ataxia	Autosomal recessive	AAG	7	9	This condition is recessive which means these are likely only carriers
FECD	Fuchs endothelial corneal dystrophy 3	Autosomal dominant	AGC	77 (6.9%)	89 (3.6%)	This is a common condition with a reported allele frequency of 3% in controls
HD	Huntington disease	Autosomal dominant	AGC	1	0	Upon testing with ExpansionHunter 3, this was determined to be a false positive
CANVAS	Cerebellar ataxia with neuropathy and bilateral vestibular areflexia syndrome	Autosomal recessive	AAGGG	35	0	This condition is recessive which means these are likely only carriers

For each disease, the inheritance, repetitive motif, and number of samples in each cohort with TRs long enough to be potentially pathogenic as predicted by EHDn IRR counts are given. An explanation is also included about the typicality and likely pathogenicity of each result.

Four of the disease loci detected are for disorders caused by the expansion of a mutated motif of the reference TR at that locus (reference motif only). The samples all fall within the previously reported distributions of allele sizes for each of these diseases. Finally, we found motifs at the loci for spinocerebellar ataxia 31 (SCA31) and FRDA which are not the disease motif nor the reference TR motif. Two of these alternative motifs for SCA31 have been previously reported in the literature, whilst the third, AAACT, is novel^42^. Additionally, the alternative motif seen at the locus for FRDA is novel as well. We also found 11 different motifs at the locus for CANVAS, indicating its highly polymorphic nature (Supplementary Figure 5). Due to the current scarcity of data on repeat sequence variation in controls, it is expected that as more genomes are analyzed, the ranges and allele frequency distributions will be modified.

### Functional *in silico* analysis of genes containing large TRs

We used PANTHER’s gene list analysis to organize genes with large TRs into their functional categories. The most common subcellular localization of proteins encoded by these genes are organelles and membranes (Supplementary Figure 7a). Forty percent of the genes are involved in binding and 30% have catalytic activity (Supplementary Figure 7b). We also conducted Fisher’s exact tests to determine whether there is an overrepresentation of genes involved in certain biological processes. After Bonferroni correction, we found that the majority of significantly enriched terms which showed over 2-fold enrichment are involved in neuronal processes (Supplementary Figure 7c,d,f). This is not the case when we do not select for repeats larger than 180 bp in the hg19 reference genome (Supplementary Figure 7e). This is an interesting observation due to the link between repeat expansions and neurological disorders. It seems that genes involved in neuronal pathways tend to have larger repeats even in control populations.

## Discussion

Here, we present a detailed characterization of an unbiased genome-wide search for TRs larger than ~175 bp. We used a set of 1,115 WGS samples to demonstrate that individual genomes typically harbor several rare large TRs and that most observed large TRs are rare, occurring in less than 1% of samples. These rare, large TRs show clear enrichment for being proximal to Alu elements and clear depletion for occurrence in exonic regions. Arcot *et al*. suggest three potential mechanisms for the proximity of TRs and *Alu* elements. First, Alus may integrate within a preexisting TR during transposition, thus resulting in an Alu flanked by repeats. Second, during reverse transcription of the *Alu*, mutations that initiate and expand a TR may occur. And third, mutations may occur in the polyA tail of the *Alu*, thus generating a TR^43^. The vast majority of the large TRs we found are expansions of smaller TR sequences documented in the hg19 reference genome. Inspection of known pathogenic loci revealed some unexpected variation, but for the most part still within known benign limits. This work establishes a database of large TR variation that can be used to help filter out non-pathogenic expansions in patients with a disorder suspected to be caused by a novel TR expansion.

In terms of evolution, the enhanced cognitive abilities of humans are thought to be a result of more sophisticated gene regulation as opposed to the number of genes which does not vary drastically between species. Several studies have implicated STRs in gene expression regulation. Rodriguez *et al*. show that the CCG repeat in the 5′UTR of FMR1 – expansions of which lead to Fragile X – is involved in regulating the translation of the FMR protein at normal repeat sizes^44^. Moreover, Fotsing *et al*. describe how the number of repeats in STRs is linearly correlated with phenotypic expression of complex traits. The contribution of STRs to phenotypic variation is greater than SNPs due to their multiallelic nature^45^. It seems intuitive that genes involved in neuronal pathways would be intrinsically diverse between individuals, due to the vast array of cognitive and behavioral capacities among the human population. Finally, it has been demonstrated that longer repeats are more polymorphic and less conserved with chimpanzees than shorter repeats, which could explain why we see an overrepresentation of genes in neuronal pathways only in datasets looking at larger repeats^46^.

Numerous prior studies have similarly characterized the TR landscape, but our approach overcomes key technical challenges that allow us new insight and is much more thorough than prior analyses performed with comparable methodologies. In 2012, Gymrek *et al*. used LobSTR to characterize the TR landscape of the 1000 Genomes samples^17^. However, LobSTR is only able to operate at pre-defined loci and can only genotype TR alleles shorter than the read length. As a result, catalogues of TR variation created with LobSTR (as well as HipSTR and other such tools)^18^ can be viewed as complementary to ours as we exclusively examine sites longer than the read length.

In 2019, Mousavi *et al*. similarly characterized the genomes of 150 individuals using GangSTR. GangSTR is able to genotype sites greater than the read length but requires designation of a set of reference TR sites to inspect^16^. While this approach undoubtedly encompasses the vast majority of TRs, it is still blind to TRs that fall outside those pre-defined sites as well as variants of the pre-defined TRs with non-referent motif patterns. In this study, we observed approximately 12% of all large TRs did not match any reference TR site, which suggests that tools such as GangSTR would have missed those expansions, although this was not directly tested. A notable example of such an exception is in the expanded and mutated motif observed in RFC1 intron 2 in some patients with CANVAS^19^. Interestingly, in Mousavi *et al*.’s analysis of 150 WGS samples, they noted that they only observed approximately 6 TRs per person with at least one allele longer than 150 bp^16^. This is surprisingly low compared to the ~260 such events we observed per genome. This discrepancy is much greater than what is predicted due to the reference TR site bias discussed above and warrants further investigation.

The most similar analysis to ours that we know of was performed by Dashnow *et al*. using the STRetch method. STRetch is very similar to the ExpansionHunter Denovo method in that both are unconstrained by read length or target site designations^20^. Dashnow *et al*. applied STRetch to a cohort of 97 PCR-free WGS samples, but their discussion of the results was restricted to an analysis analogous to what we showed in our Fig. 6.

The authors of TREDPARSE analyzed far more WGS samples than us but restricted their analysis to a relatively small number of predefined trinucleotide repeat loci. They used the TREDPARSE software to estimate repeat sizes at 30 different disease loci in 12,632 individuals. Their approach also revealed asymptomatic individuals with potentially pathogenic expansions at several disease loci tested. However, loci composed of GC rich motifs lacked sufficient read evidence^21^. In our study, we also see a bias against GC rich motifs at 0.57% of our dataset, but this is in alignment with the percentage of GC rich motifs in the hg19 reference genome. Nevertheless, there is concern regarding a sequencing bias against GC rich regions of the genome in both these cases.

A major limitation of our approach is that allele sizes are not actually estimated. We merely get counts of anchored IRRs endorsing each TR for each sample. This makes comparisons to results with other tools unintuitive and new samples analyzed with EHDn need to be normalized according to read depth before they can be directly compared either. This lack of allele sizing also means that our database does not resolve whether a sample has just one allele longer than the read length or both. This increases difficulty of interpretation, particularly for recessive disease samples. A third limitation of our approach is that the number of reads reported by EHDn endorsing any given expansion event will saturate for expansions over a certain size due to the fact that this approach can only use anchored IRRs and not IRR pairs. A fourth limitation is that the cohort is almost entirely white, non-Hispanic, which certainly lends some systematic bias to our results. However, preliminary analysis of healthy samples of diverse ethnicities from the 1000 Genomes Project show similar distributions compared to our cohort, which relieves some of these concerns (Supplementary Figures 1–3). While long read sequencing would be ideal for studying structural variation, the technology is significantly more expensive than short read sequencing. Additionally, a large number of samples have already been sequenced using short read technology, so it is important that we are able to extract the relevant mutations from these valuable resources instead of investing additional money into subjecting them to long read sequencing. When comparing our data with PacBio long read sequence, we find that 96% of the TRs identified by EHDn are indeed longer than 175 bp, which means we have a very low false positive detection rate (Supplementary Table 3). Hopefully, future studies will be able to alleviate and rectify the limitations discussed here through development of more sophisticated tools and application to larger, more diverse sample sets.

## Methods

### VAFR samples processing

The 1,115 PCR-free whole genome sequencing samples used in this study were generated as part of the NHLBI TOPMed Vanderbilt Atrial Fibrillation Registry. Prior to submission to dbGaP, the TOPMed Informatics Research Center uniformly processed the samples from the Vanderbilt Atrial Fibrillation Registry. They aligned the fastq files to the 1000 Genomes hs37d5 build 37 decoy reference sequence using BWA-mem version 0.7.12-r1039 with ‘-M’ to mark split alignments. This was followed by ‘samtools sort’, ‘bamUtils polishBam’, and ‘bamUtils dedup_LowMem --recab’ with flags ‘--binMid --binQualS 2,3,10,20,25,30,35,40,50 --maxBaseQual 44’ to recalibrate and bin base call quality scores. These processed bam files were then retrieved from dbGaP Study Accession phs001032.v3.p2 as Short Read Archive (SRA) files, at which point the sam alignment was extracted using the SRA Toolkit command ‘sam-dump -u’, and marked duplicates were removed using ‘samtools view’ with parameters ‘-b -F 1024’. Samtools version 1.2 was used. ExpansionHunter Denovo^15^ version 0.6.2 was then run on these files continuing to use the hs37d5 reference sequence with additional parameters ‘--min-unit-len 3 --max-unit-len 8’ to restrict the search to motifs of length 3 to 8 nucleotides long.

### 1000 Genomes Project samples processing

We also utilized PCR-free whole genome sequencing samples produced by the 1000 Genomes Project. We downloaded the high coverage WGS samples as cram files from ftp://ftp-trace.ncbi.nlm.nih.gov/1000genomes/ftp/1000G_2504_high_coverage/. These samples had been sequenced to ~30X coverage and aligned to the 1000 Genomes project version of GRCh38 (GRCh38DH) using BWA-mem version 0.7.15, duplicate reads marked using Picard version 2.4.1, and base quality scores recalibrated using GATK version 3.5.0, as fully described here: ftp://ftp.1000genomes.ebi.ac.uk/vol1/ftp/data_collections/1000G_2504_high_coverage/20190405_NYGC_b38_pipeline_description.pdf. Similar to the TOPMed samples described above, ExpansionHunter Denovo was then run on these cram files using GRCh38DH as the reference sequence with the additional parameters ‘--min-unit-len 3 --max-unit-len 8’ to restrict the search to motifs of length 3 to 8 nucleotides long. The repetitive region calls were later converted to GRCh37 coordinates using the UCSC liftOver utility prior to performing the analyses shown in the figures.

### ExpansionHunter Denovo (EHDn) processing

The outputs of EHDn for each of the 1,115 samples were then aggregated using EHDn’s helper scripts ‘combine_counts.py’ and ‘compare_anchored_irrs.py’ to allow comparisons among the samples in a read depth-normalized manner. Finally, the bed file output by the ‘compare_anchored_irrs.py’ script was then expanded from its sparse encoding into a dense matrix format using R. This dense matrix of depth-normalized anchored IRR counts was used as the input for analyses in this manuscript and is provided in Supplementary Table 1^22^. This process was repeated for the 2,504 samples from the 1000 Genomes project to create Supplementary Table 2^22^. The scripts used to process the data have been deposited in a Github repository: [https://github.com/ZuchnerLab/Fazal2020Scripts].

### PacBio long read data processing

PacBio HiFi long read data produced by the Human Genome Structural Variation Consortium for five of the samples from the 1000 Genomes cohort were downloaded from ftp://ftp.1000genomes.ebi.ac.uk/vol1/ftp/data_collections/HGSVC2/working/. For each sample, the circular consensus sequence fastq files were downloaded. Fastq files were mapped to the hs37d5 reference genome using Minimap2-2.17 using the parameters “--MD -ax asm20”. The mapped reads were then converted to bam files and sorted using Sambamba version v0.5.9.

### Saturation curves

Number of novel TRs with supporting reads above 5 was counted for each genome in the cohort. To avoid bias in the order in which the samples were processed, 1000 random iterations were done, each with a different order for sample processing. Averages of the number of novel TRs expected were calculated from the 1000 iterations, and these values were used to plot the cumulative number of TRs as a function of sample size. To estimate how many novel TRs were still expected to be observed after processing of the 1,115 genomes in our cohort, we averaged the estimates for the last 15 genomes processed.

### Annotation and normalization to genomic regions

Homer software v4.10 was used to annotate the TRs based on their chromosomal positions. The Homer data annotation file was separated according to the genomic regions; intergenic, intron, promoter, exon, TTS, and also according to *Alu* overlapping and non-overlapping regions. Bedtools fisher (v2.29.0) was used to perform a Fisher’s exact test of overlap between two sets of chromosomal locations. The TRs discovered in our cohort were tested against each set of genomic regions to get an odds ratio, which determines whether the degree of overlap observed is more or less than expected given the size of the genome and the proportion of the genome each region comprises. The same was done for simple repeats from the hg19 reference genome.

### Reference repeat loci overlap

The Simple Repeats table for the hg19 reference genome was downloaded from the UCSC Genome Browser website. These were filtered to only include motifs between 3 and 8 base pairs in length as this is comparable to our set of TRs. Homer software was used to annotate the repeats in order to categorize them into the different groups. Bedtools intersect (v2.17.0) was used to create an output file containing TRs that overlapped the chromosomal position of a simple repeat in the reference genome. This was then filtered for matching motifs, and reverse complements were considered to be a match. Duplicate TRs, or those that matched multiple simple repeats, were removed and the resulting TRs were considered to have originated from a reference TR site. Percentages were calculated for each subcategory.

### Read and repeat length correlation analysis

ExpansionHunter was run on a subset of samples at specific cataloged sites in the genome. These were at known disease loci detailed in Table 2, and the 11 CANVAS motifs (Supplemental Figure 3). Only 5 of the loci were selected as they contained a large enough sample size; SCA37, SCA31 (AAAAT), CANVAS (AAAAG), CANVAS (AAAGG), and CANVAS (AAGGG). The output contains estimated allele sizes at each locus for each sample. These were plotted against the number of reads estimated by ExpansionHunter Denovo, and Spearman rho correlation values were calculated for each locus tested. Results produced correlation values between 0.67 and 0.86, which demonstrates strong correlation between the anchored IRR counts and the larger allele estimated by ExpansionHunter. Based on the slopes of the correlation plots, an average equation was produced to estimate the number of bases in the repeat region, which can be used to calculate the number of repeats based on the read count. This equation was used to estimate allele lengths for samples which were not run with ExpansionHunter. The equation used is *y = 140.6* + *7.7x*, where *y* is the number of bases and *x* is the number of reads. See Supplementary Figure 2.

## Supplementary information

Supplementary materials

## Data Availability

All data generated or analyzed during this study are included in this published article (and its Supplementary Information Files), in the figshare database(10.6084/m9.figshare.c.4819050)^22^, or in the associated Github repository (https://github.com/ZuchnerLab/Fazal2020Scripts).

## Code availability

Source code can be found in the Github repository: (https://github.com/ZuchnerLab/Fazal2020Scripts).
