# Supplementary material for: Large scale in silico characterization of repeat expansion variation in human genomes
Source: Sci Data. 2020 Sep 8;7:294. doi: 10.1038/s41597-020-00633-9 (PMC7479135; doi:10.1038/s41597-020-00633-9)
Supplement: Supplementary file 1 — Supplementary materials [file 41597_2020_633_MOESM1_ESM.pdf]

Table of Contents

Supplementary figure 1 ……………………………………………………………………………………………………Page 1
Supplementary figure 2 ……………………………………………………………………………………………………Page 2
Supplementary figure 3 ……………………………………………………………………………………………………Page 3
Supplementary figure 4 ……………………………………………………………………………………………………Page 4
Supplementary figure 5 ……………………………………………………………………………………………………Page 5
Supplementary figure 6 ……………………………………………………………………………………………………Page 6
Supplementary figure 7 ……………………………………………………………………………………………………Page 7
Supplementary table 3 ……………………………………………………………………………………………Page 8-13
Supplementary table 4 ……………………………………………………………………………………………Page 14-16
Supplementary material legends ……………………………………………………………………………Page 17-18

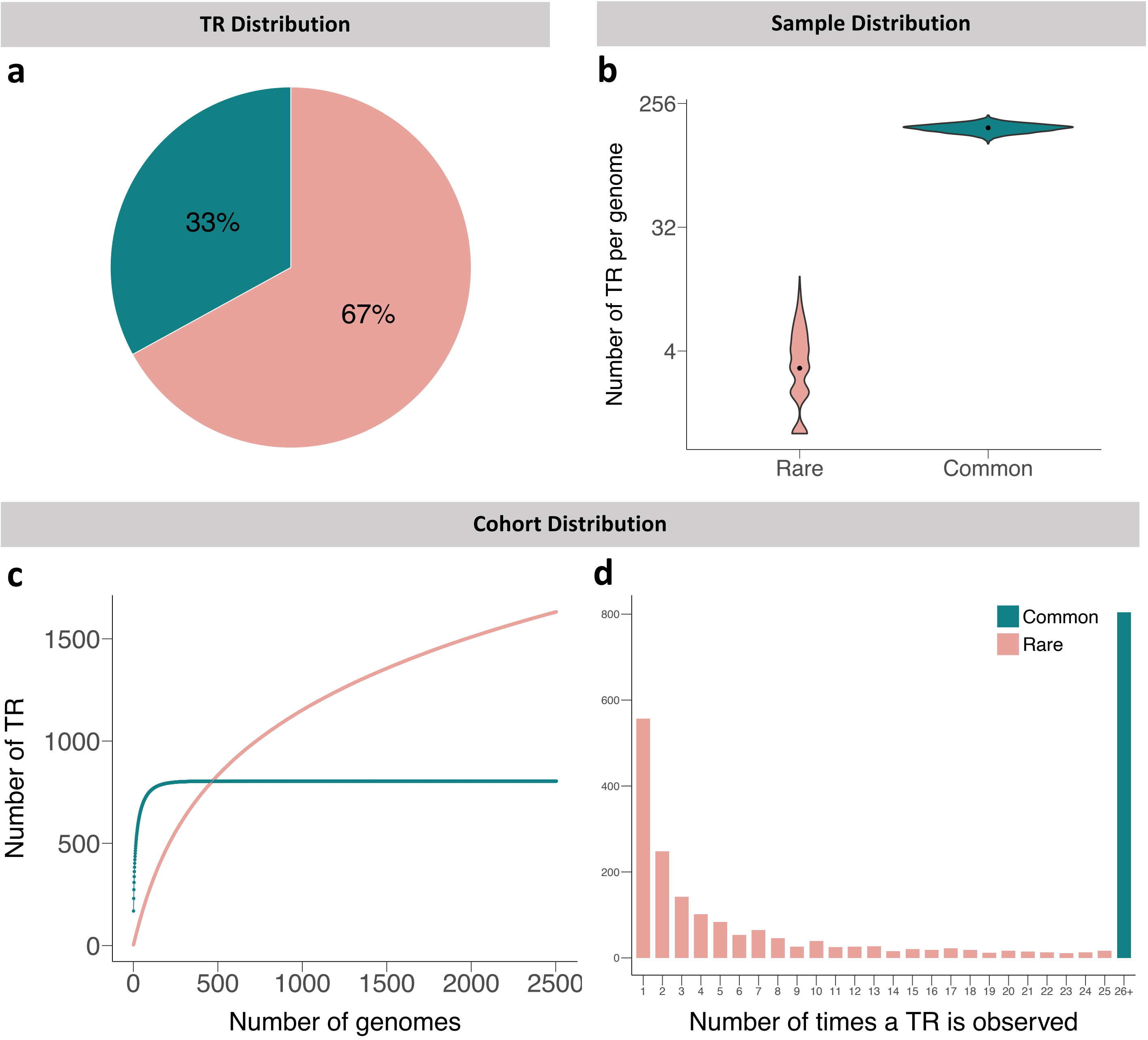
TR Distribution
a
33%
67%
Sample Distribution
b
Number of TR per genome
256
32
4
Rare
Common
Cohort Distribution
c
Number of TR
1500
1000
500
0
0
500
1000
1500
2000
2500
Number of genomes
d
Common
Rare
800
600
400
200
0
1 2 3 4 5 6 7 8 9 10 11 12 13 14 15 16 17 18 19 20 21 22 23 24 25 26+
Number of times a TR is observed

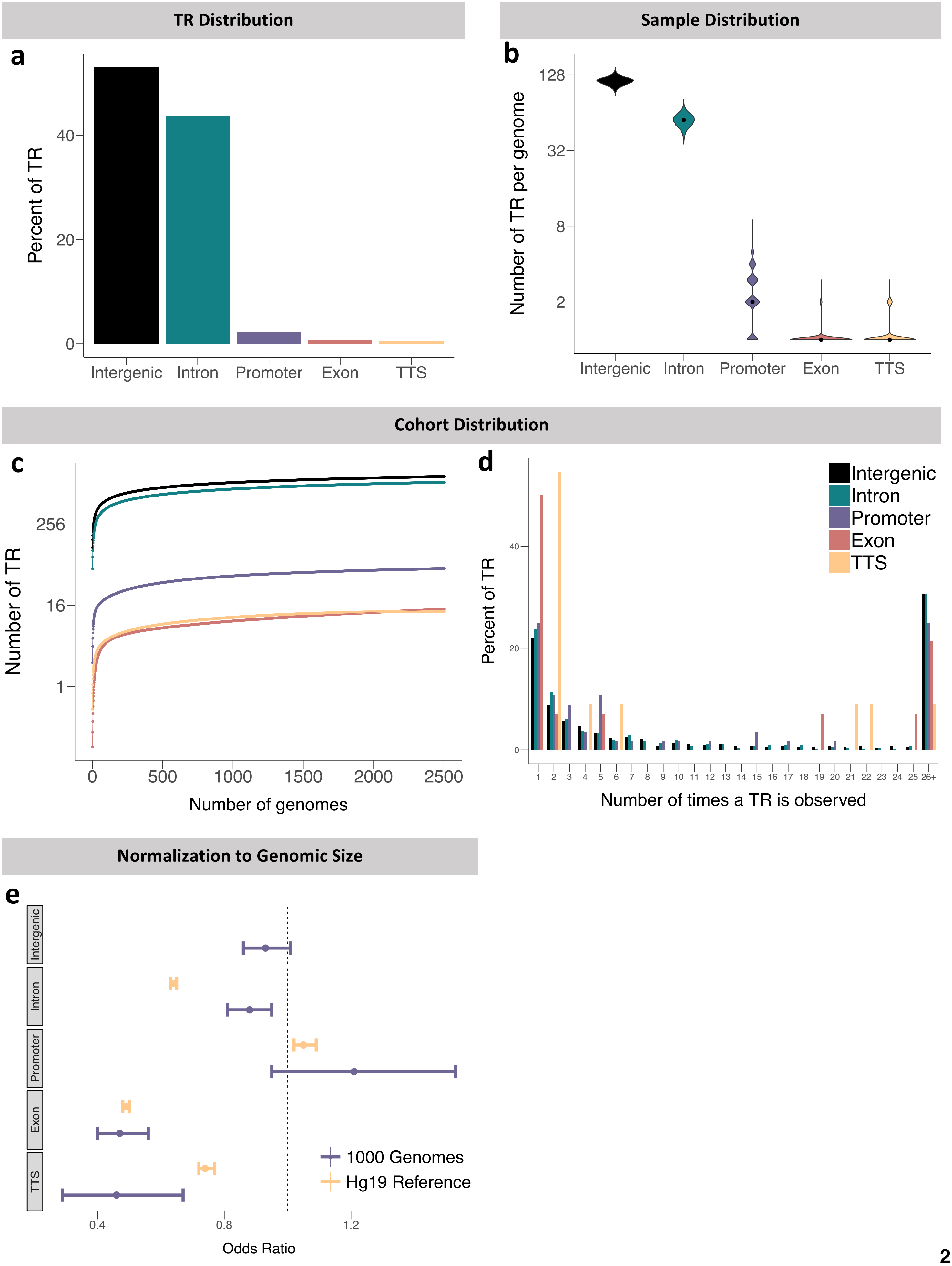
TR Distribution
a
Percent of TR
0
20
40
Intergenic
Intron
Promoter
Exon
TTS
Sample Distribution
b
Number of TR per genome
2
8
32
128
Intergenic
Intron
Promoter
Exon
TTS
Cohort Distribution
c
Number of TR
1
16
256
0
500
1000
1500
2000
2500
Number of genomes
d
Percent of TR
0
20
40
Intergenic
Intron
Promoter
Exon
TTS
1 2 3 4 5 6 7 8 9 10 11 12 13 14 15 16 17 18 19 20 21 22 23 24 25 26+
Number of times a TR is observed
Normalization to Genomic Size
e
Intergenic
Intron
Promoter
Exon
TTS
1000 Genomes
Hg19 Reference
0.4
0.8
1.2
Odds Ratio

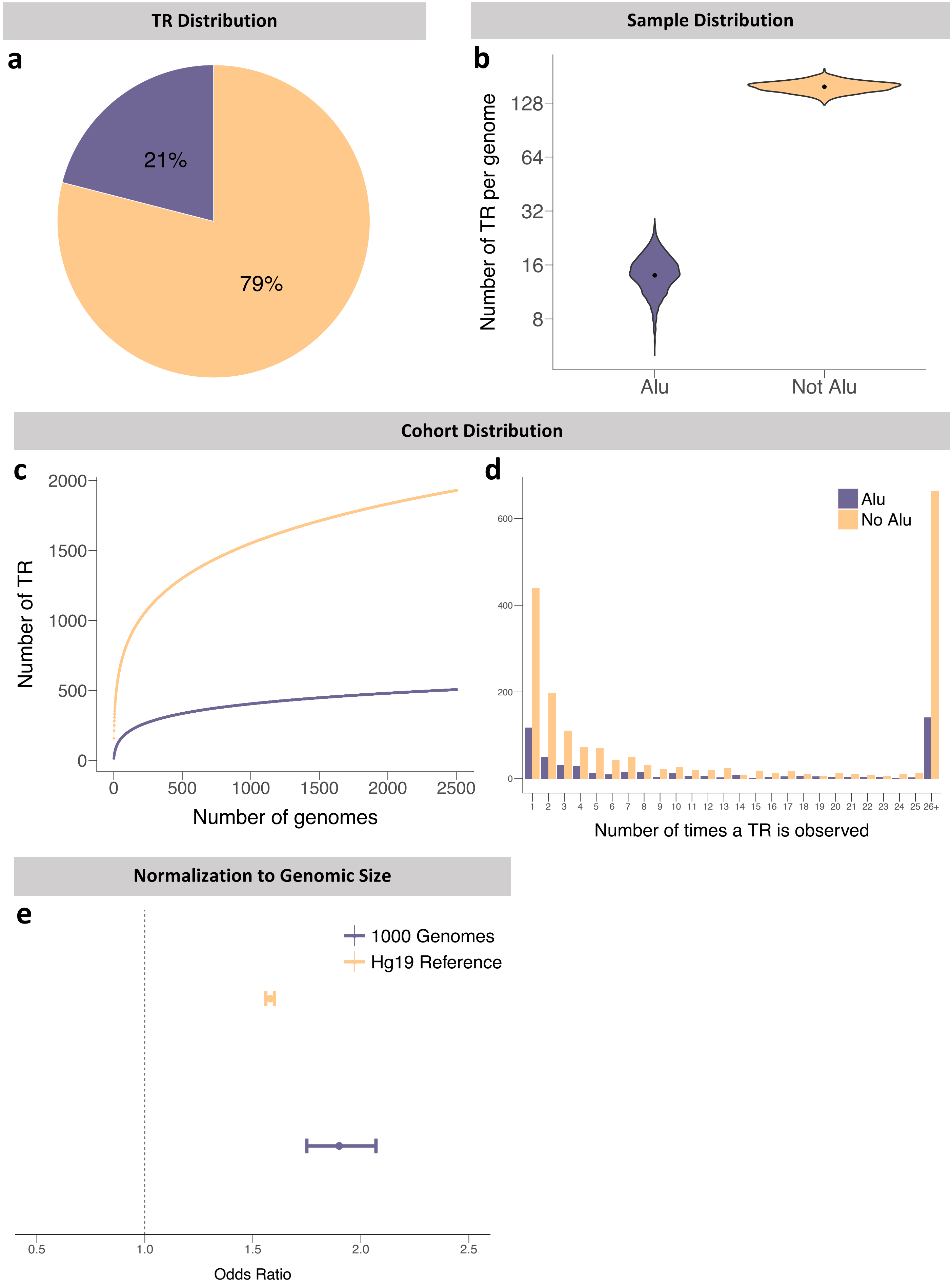
TR Distribution
a
21%
79%
Sample Distribution
b
Number of TR per genome
128
64
32
16
8
Alu
Not Alu
Cohort Distribution
c
Number of TR
2000
1500
1000
500
0
0
500
1000
1500
2000
2500
Number of genomes
d
600
400
200
0
Alu
No Alu
1 2 3 4 5 6 7 8 9 10 11 12 13 14 15 16 17 18 19 20 21 22 23 24 25 26+
Number of times a TR is observed
Normalization to Genomic Size
e
1000 Genomes
Hg19 Reference
0.5
1.0
1.5
2.0
2.5
Odds Ratio

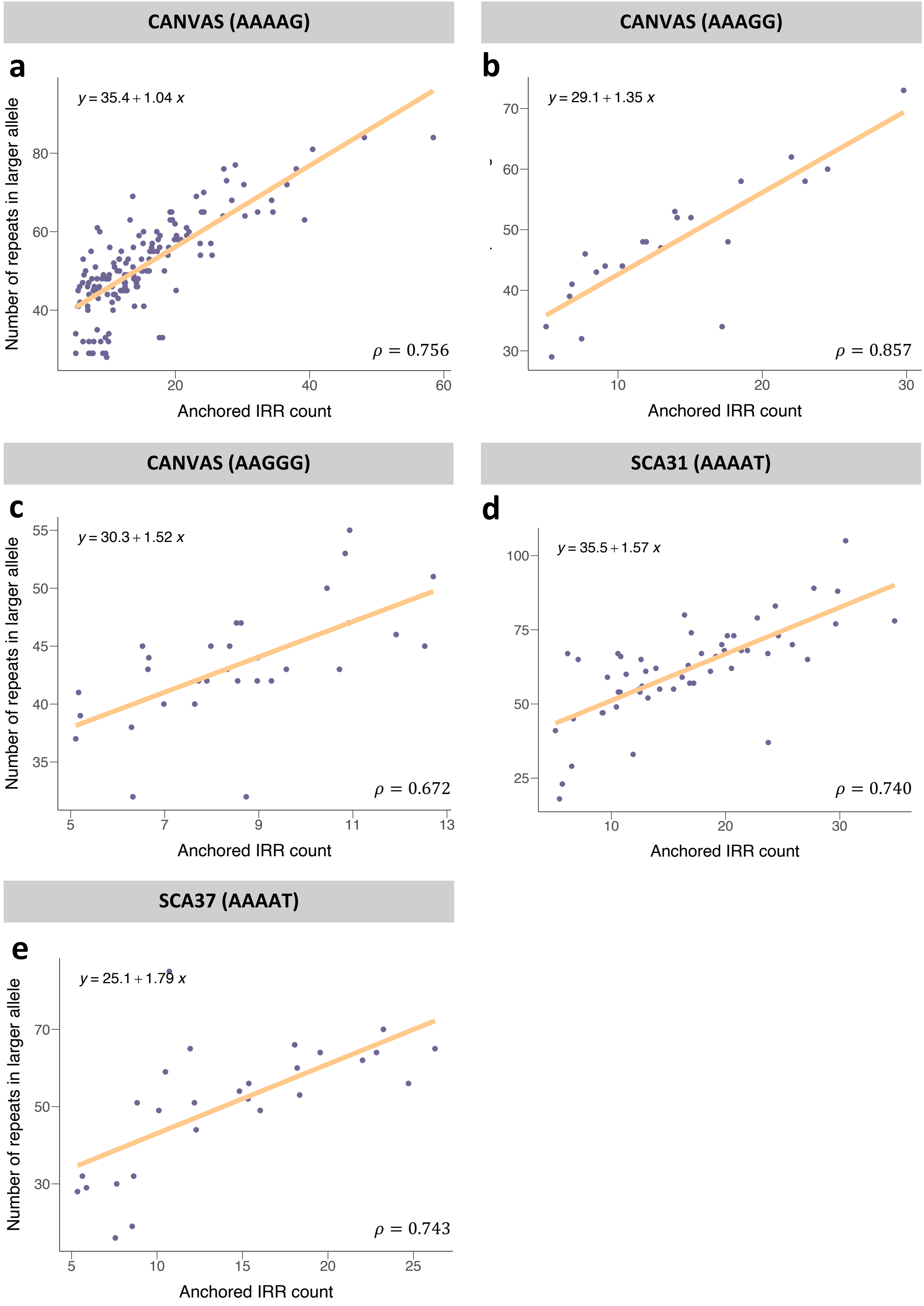
CANVAS (AAAAG)
a
y = 35.4 + 1.04 x
ρ = 0.756
Number of repeats in larger allele
Anchored IRR count
CANVAS (AAAGG)
b
y = 29.1 + 1.35 x
ρ = 0.857
Anchored IRR count
CANVAS (AAGGG)
c
y = 30.3 + 1.52 x
ρ = 0.672
Number of repeats in larger allele
Anchored IRR count
SCA31 (AAAAT)
d
y = 35.5 + 1.57 x
ρ = 0.740
Anchored IRR count
SCA37 (AAAAT)
e
y = 25.1 + 1.79 x
ρ = 0.743
Number of repeats in larger allele
Anchored IRR count

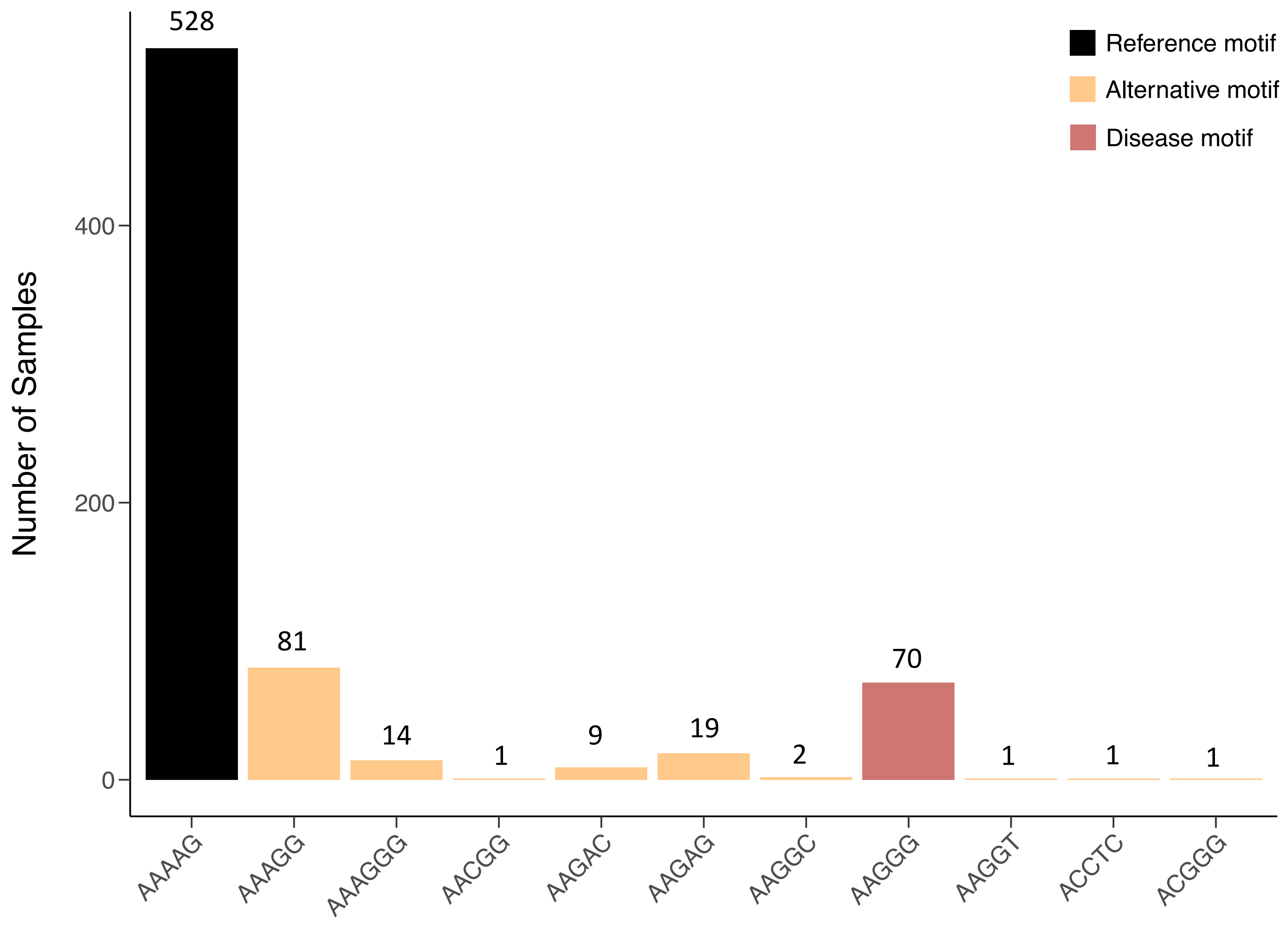

528
81
14
1
9
19
2
70
1
1
1
Reference motif
Alternative motif
Disease motif
Number of Samples
400
200
0
AAAAG
AAAGG
AAAGGG
AACGG
AAGAC
AAGAG
AAGGC
AAGGG
AAGGT
ACCTC
ACGGG

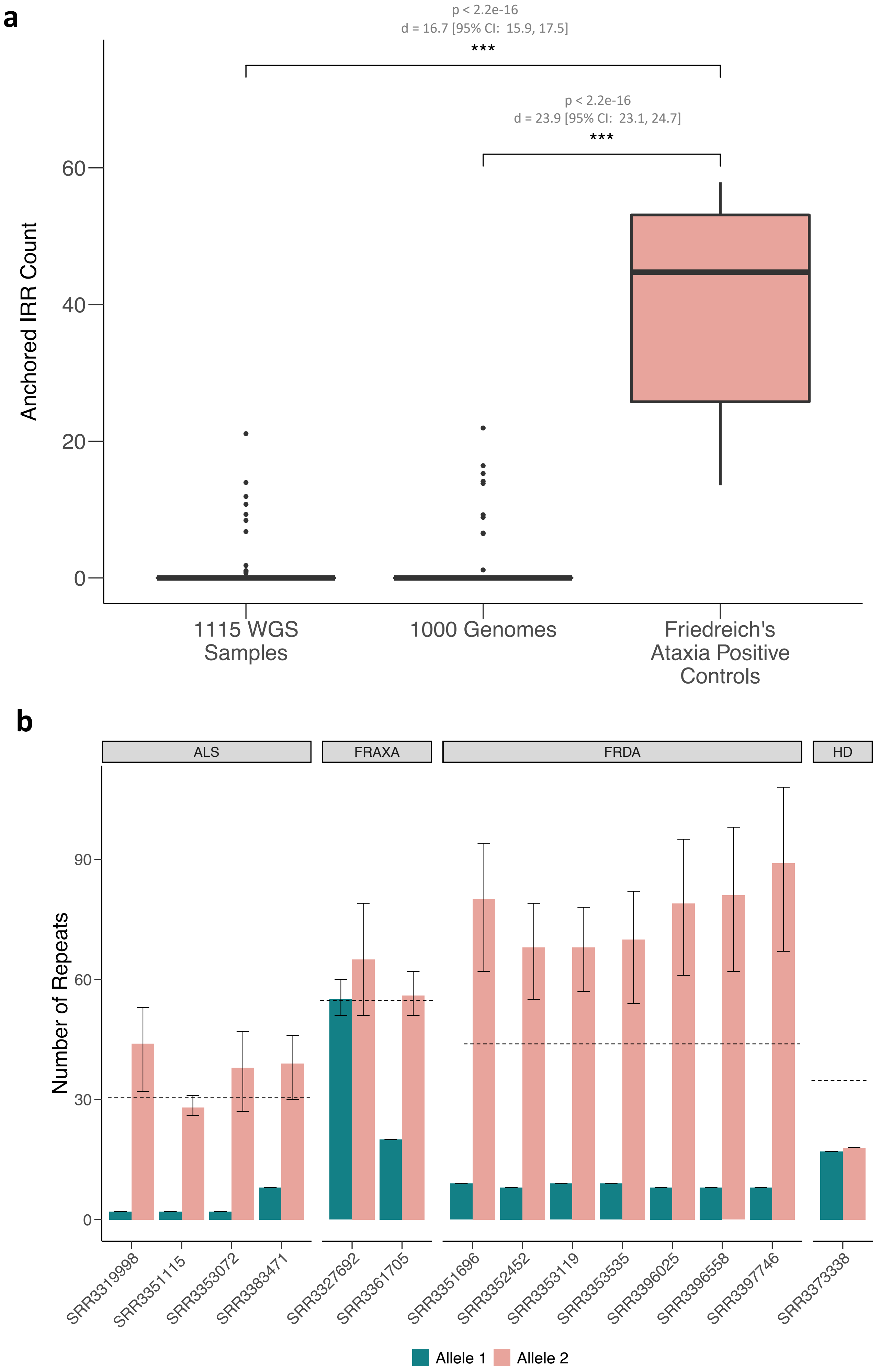
a
p < 2.2e-16
d = 16.7 [95% CI: 15.9, 17.5]
***
p < 2.2e-16
d = 23.9 [95% CI: 23.1, 24.7]
***
Anchored IRR Count
0
20
40
60
1115 WGS Samples
1000 Genomes
Friedreich's Ataxia Positive Controls
b
ALS
FRAXA
FRDA
HD
Number of Repeats
0
30
60
90
SRR3319998
SRR3351115
SRR3353072
SRR3383471
SRR3327692
SRR3361705
SRR3351696
SRR3352452
SRR3353119
SRR3353535
SRR3396025
SRR3396558
SRR3397746
SRR3373338
Allele 1
Allele 2

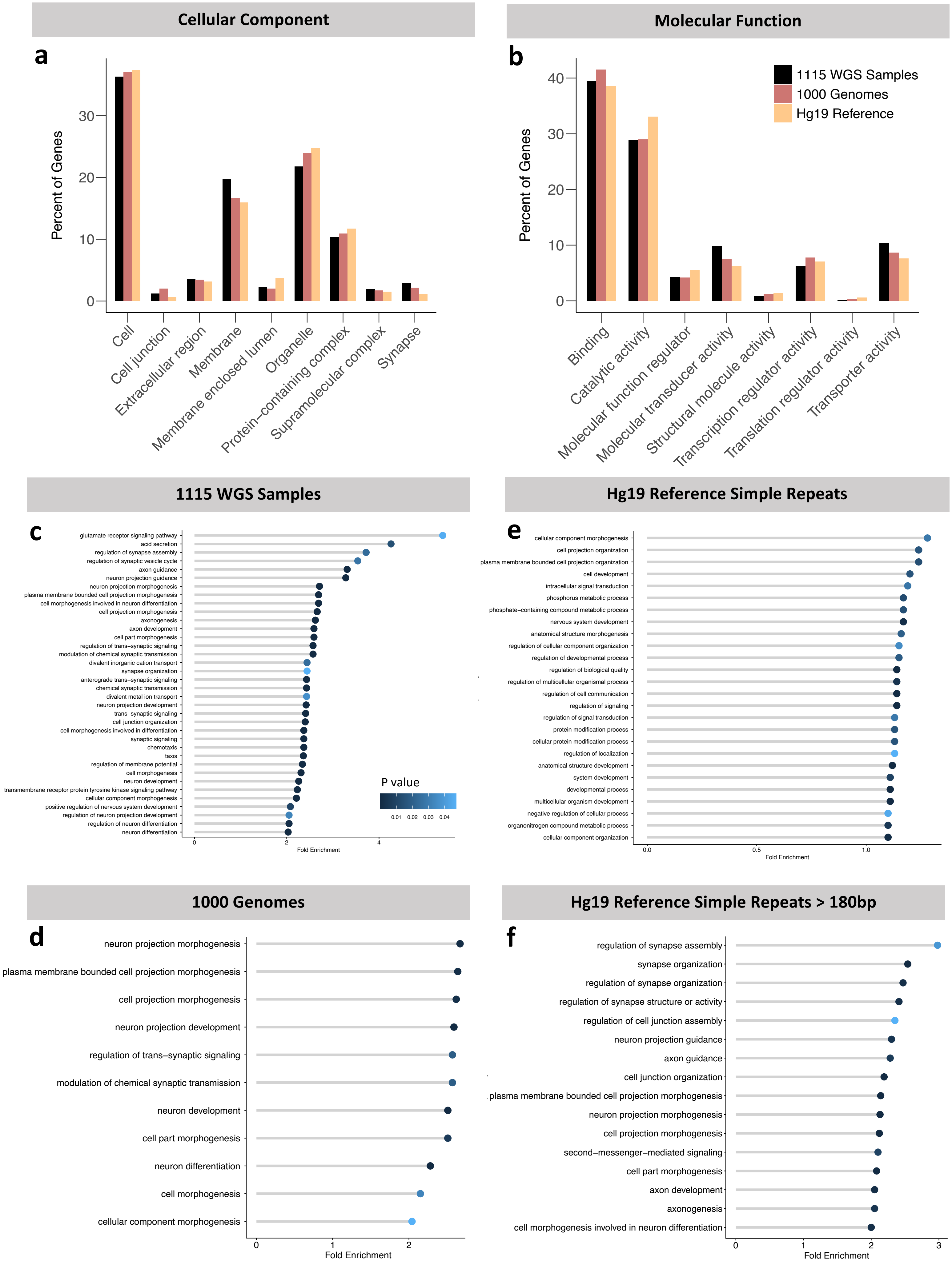
Cellular Component
a
Percent of Genes
0
10
20
30
Cell
Cell junction
Extracellular region
Membrane
Membrane enclosed lumen
Organelle
Protein−containing complex
Supramolecular complex
Synapse
Molecular Function
b
Percent of Genes
0
10
20
30
40
1115 WGS Samples
1000 Genomes
Hg19 Reference
Binding
Catalytic activity
Molecular function regulator
Molecular transducer activity
Structural molecule activity
Transcription regulator activity
Translation regulator activity
Transporter activity
1115 WGS Samples
c
glutamate receptor signaling pathway
acid secretion
regulation of synapse assembly
regulation of synaptic vesicle cycle
axon guidance
neuron projection guidance
neuron projection morphogenesis
plasma membrane bounded cell projection morphogenesis
cell morphogenesis involved in neuron differentiation
cell projection morphogenesis
axonogenesis
axon development
cell part morphogenesis
regulation of trans−synaptic signaling
modulation of chemical synaptic transmission
divalent inorganic cation transport
synapse organization
anterograde trans−synaptic signaling
chemical synaptic transmission
divalent metal ion transport
neuron projection development
trans−synaptic signaling
cell junction organization
cell morphogenesis involved in differentiation
synaptic signaling
chemotaxis
taxis
regulation of membrane potential
cell morphogenesis
neuron development
transmembrane receptor protein tyrosine kinase signaling pathway
cellular component morphogenesis
positive regulation of nervous system development
regulation of neuron projection development
regulation of neuron differentiation
neuron differentiation
0
2
4
Fold Enrichment
P value
0.01 0.02 0.03 0.04
Hg19 Reference Simple Repeats
e
cellular component morphogenesis
cell projection organization
plasma membrane bounded cell projection organization
cell development
intracellular signal transduction
phosphorus metabolic process
phosphate−containing compound metabolic process
nervous system development
anatomical structure morphogenesis
regulation of cellular component organization
regulation of developmental process
regulation of biological quality
regulation of multicellular organismal process
regulation of cell communication
regulation of signaling
regulation of signal transduction
protein modification process
cellular protein modification process
regulation of localization
anatomical structure development
system development
developmental process
multicellular organism development
negative regulation of cellular process
organonitrogen compound metabolic process
cellular component organization
0.0
0.5
1.0
Fold Enrichment
1000 Genomes
d
neuron projection morphogenesis
plasma membrane bounded cell projection morphogenesis
cell projection morphogenesis
neuron projection development
regulation of trans−synaptic signaling
modulation of chemical synaptic transmission
neuron development
cell part morphogenesis
neuron differentiation
cell morphogenesis
cellular component morphogenesis
0
1
2
Fold Enrichment
Hg19 Reference Simple Repeats > 180bp
f
regulation of synapse assembly
synapse organization
regulation of synapse organization
regulation of synapse structure or activity
regulation of cell junction assembly
neuron projection guidance
axon guidance
cell junction organization
plasma membrane bounded cell projection morphogenesis
neuron projection morphogenesis
cell projection morphogenesis
second−messenger−mediated signaling
cell part morphogenesis
axon development
axonogenesis
cell morphogenesis involved in neuron differentiation
0
1
2
3
Fold Enrichment

Rare or Common	EHDn Chr	EHDn Start	EHDn End	EHDn motif	EHDn IRRCount	SimpleRepeat Overlaps	SimpleRepeat Size	SimpleRepeat Motif	PacBio Allele1 Size	PacBio Allele1 Motif	PacBio Allele2 Size	PacBio Allele2 Motif	Sniffles Call	Notes
**ID #NA19238**														
Common	10	34954246	34955192	AACAG	17.3	Yes	90	AACAG	95	AACAG	~2070	AACAG	Allele 2 insertion	
Common	11	57170302	57171718	AAG	6.1	Yes	216	AAG	~275	AAG	~290	AAG	Allele 1 insertion	Sniffles call is just a ~245bp site
Common	12	9373280	9374830	AAAG	19.9	No	-	-	230	AAAG	260	AAAG	Allele 2 insertion	No simple repeats table entry, but there is a small repetitive sequence of AAAG in the ref (40bp)
Common	14	100418080	100420071	ACC	8.6	Yes	78	ATGGTG	~765	ACC	~1075	ACC	Allele 1 insertion	
Common	17	72478103	72479368	AAAG	16.4	Yes	28	AAGG	~460	AAGG and AAAG	~560	AAGG and AAAG	None	
Common	2	11093033	11095028	ACATCC	9.5	No	-	-	~880	ACATCC	~1580	ACATCC	None	Expansion motifs are relatively impure. Seed site also isn't readily apparent
Common	3	128305037	128306625	AAAGG	44.1	Yes	125	AAAGG	415	AAAGG	unclear	-	None	Most reads end in the repetitive region. The second allele could be another much larger expansion (or it could be a difficult area to sequence)

Common	6	119061784	119063279	AAAAT	19.9	Yes	70	AAAAT	70	AAAAT	340	AAAAT	Allele 2 insertion	
Common	8	9139955	9141524	AAAGG	14.7	Yes	120	AAAGG	~260	AAAAGG	unclear	-	None	Low coverage. Difficult to tell if the two alleles are similar in size or if only one is being detected.
Common	9	78965722	78967102	AAAG	6.1	Yes	240	AAAG	~267	AAAG	~340	AAAG	None	
**ID #NA19239**														
Rare	10	26163426	26163999	AAGGG	5.5	Yes	250	AAGGG	248	AAGGG	258	AAGGG	None	
Rare	10	120007233	120008182	AAAGAGAG	10.0	Yes	120	AAGG	~250	very impure -- all As and Gs	~250	very impure -- all As and Gs	None	Can't tell two PacBio alleles apart; low coverage
Rare	17	2017630	2018803	AAAAT	7.7	Yes	100	AAAAT	153	AAAAT	~4300	AAAAT	None	
Rare	17	20459646	20460751	ACGGGAG	8.8	Yes	150	AGAGGG	35	AGAGGG	193	ACGGGAG	Allele 1 deletion	
Rare	19	5795579	5796806	AAGGAG	8.8	Yes	175, 50, 10	AGG, AAG, AAG	10	AAG	~270	AAGGAG	Allele 1 deletion	
Rare	4	4264999	4265852	AAAG	15.5	No	-	-	27	AAAG	~410	AAAG	None	No simple repeats table entry, but there is a small repetitive sequence of AAAG in the ref (24bp)
Rare	6	120781077	120782070	AAAAT	14.4	No	-	-	~125	AAAAT	~900	AAAAT	Allele 1 insertion	No simple repeats table entry, but there is a small repetitive sequence of AAAAT in the ref (50bp)

Rare	7	31373292	31374343	AAAG	7.7	Yes	80	AAAG	90	AAAG	~240	AAAG	None	
Common	11	57170302	57171718	AAG	8.8	Yes	216	AAG	181	AAG	~290	AAG	None	
Common	7	96941243	96942633	AAAG	7.7	Yes	180	AAAG	204	AAGG	234	AAAG	None	
**ID #HG00513**														
Common	1	31248331	31249883	AAAGG	13.0	Yes	180	AAAGG	195	AAAGG	205	AAAGG	None	PacBio alleles show deletions of about 80 and 90bp within a large, impure AG-rich region of approximately 285bp (in which lies the 180bp simple repeats table site, which has a pure motif pattern)
Common	2	97324043	97326063	AAAG	9.1	Yes	180, 40	AAAG, AAGG	180	AAAG	~620	AAAG	None	
Common	2	26810930	26812585	AAG	5.7	Yes	180	AAG	180	AAG	186	AAG	None	
Common	4	102026733	102028223	AAAG	10.2	Yes	164	AAAG	200	AAAG	325	AAAG	Allele 2 insertion	
Common	5	180084613	180086784	ACAGAGGG	11.9	No	-	-	347	ACAGAGGG	~500	ACAGAGGG	Allele 2 insertion	No simple repeats table entry, but there is a repetitive sequence of ACAGAGGG in the ref (355bp) which is factored into the PacBio allele sizes
Common	7	28149194	28150762	AAAGG	26.6	Yes	180	AAAGG	~530	AAAGG	unclear	-	None	Most reads end in the repetitive region. The few that sequence completely

														through it endorse a ~530bp AAAGG sequence
Common	1	35266877	35268743	AAACC	6.8	Yes	220	AAACC	229	AAACC	235	AAACC	None	
Common	3	111498259	111500361	AAG	17.6	Yes	160	AAG	179	AAG	410	AAG	None	
Common	4	6579963	6581746	ACC	5.1	Yes	422	ACC	~1100	ACC	~1230	ACC	Both insertions	Expansion motifs are relatively impure with Ts every 4th repeat unit or so
Common	6	24150255	24151665	AAAG	8.5	Yes	180	AAAG	~250	AAAG	~280	AAAG	Allele 1 insertion	
**ID #HG00731**														
Rare	1	22686490	22687473	GGAA	5.6	Yes	145	GGAA	145	GGAA	~201	GGAA	None	
Rare	15	59273819	59274961	TTC	6.7	Yes	101	TTC	~171	TTC	~171	TTC	Expansion	Difficult to tell the two alleles apart - probably similar in size
Rare	6	120781077	120782070	TTTTA	6.7	No	-	-	25	TTTTA	~450	TTTTA	Allele 2 insertion	No simple repeats table entry, but there is a small repetitive sequence of TTTTA in the ref (50bp)
Rare	9	96633713	96634653	AAAG	10.1	No	-	-	~122	AAAG	~230	AAAG	Allele 1 insertion	No simple repeats table entry, but there is a small repetitive sequence of AAAG in the ref (32bp)
Common	6	51854016	51,855,486	GGAA	20.1	Yes	129	GGAA	~482	GGAA	~482	GGAA	one insertion	Difficult to tell the two alleles apart -

														probably similar in size
Common	4	5707224	5708577	TAGA	5.6	Yes	174	TAGA	~2359	TAGA	~3674	TAGA	None	
Common	10	118491174	118492423	AAAG	19.0	No	-	-	28	AAAG	536	AAAG	None	Only 1 read supporting the large expansion. No simple repeats table entry, but there is a small repetitive sequence of AAAG in the ref (24bp)
Common	20	36822253	36823662	TCCA	25.7	Yes	72	TCCA	~1057	TCCA	~1102	TCCA	Allele 1 insertion	
Common	3	165215925	165217305	GGAAA	6.7	Yes	157	GGAAA	~207	GGAAA	~218	AAAGG	None	
Common	12	26668498	26670048	GGAAA	10.1	Yes	171	GGAAA	~201	GGAAA	~231	AAAGG	Allele 2 insertion	
**ID #HG00732**														
Rare	19	34535035	34536300	GAA	5.5	Yes	160	GAA	160	GAA	~280	GAA	None	Allele 2 shows 3 separate insertions (same motif)
Rare	19	49684865	49686036	TCCA	5.5	Yes	540	TCCA	~588	TCCA	~588	TCCA	None	Both alleles show multiple insertions (same motif)
Rare	X	51363866	51364860	CCTTCT	6.6	Yes	180	TTC	180	TTC	~225	CCTTCT	None	
Common	2	97324043	97326063	AAAG	6.6	Yes	179	TTTC	179	TTTC	~328	TTTC	None	Low coverage (3 reads supporting allele 1, 1 read supporting allele 2)
Common	20	36822253	36823662	ATCC	28.7	Yes	72	TCCA	~1046	TCCA	~1077	TCCA	Allele 1 insertion	

Common	11	22456697	22457982	TAAAA	14.4	Yes	105	TAAAA	105	TAAAA	~843	TAAAA	Allele 2 insertion	
Common	17	43253518	43254822	TTTC	14.4	Yes	110	TTTC	102	TTTC	~450	TTTC	None	
Common	3	111498259	111500361	GAA	12.1	Yes	160	GAA	~208	GAA	~461	GAA	Allele 1 insertion	Allele 2 has only 2 supporting reads
Common	11	123327084	123328402	AAAG	9.9	No	-	-	~163	AAAG	~163	AAAG	None	No simple repeats table entry, but there is a small repetitive sequence of AAAG in the ref (20bp). Difficult to tell the two alleles apart - probably similar in size
Common	16	3247090	3248705	AGAGGG	13.3	Yes	496	GGGAGA	~946	GGGAGA	~946	GGGAGA	319bp insertion	Multiple insertions along the repeat, difficult to tell the two alleles apart

Disease	Abbreviation	Inheritance	Gene	Type	Location in gene	Repeat sequence	Start site in reference genome (hg19)	Typical normal range of repeats	Typical expansion range of repeats	ALU involvement
Amyotrophic Lateral Sclerosis / Frontotemporal Dimentia	ALS / FTD	AD	C9ORF72	Intron	Intron 1	GGGGCC	chr9:27573483	2 - 19	250 - 1600	
Baratela-Scott Syndrome	BSS	AR	XYLT1	Promoter	Intron 1	GGC	chr16:17563050	9 - 20 (Insertion, unmethylated)	118+ (methylated)	
Blepharophimosis, epicanthus inversus, and ptosis	BPES	AD	FOXL2	Coding	Exon 1	GCN	chr3:138664863	14	15 - 24	
Central hypoventilation syndrome	CCHS	AD	PHOX2B	Coding	Exon 3	GCN	chr4:41747989	< 24	>25	
Cerebellar ataxia with neuropathy and bilateral vestibular areflexia syndrome	CANVAS	AR	RFC1	Intron	Intron 2	AAGGG	chr4:39350045	15 - 200 (reference motif is AAAAG)	40 - 1000	AluSx3
Cleidocranial dysplasia	CCD	AD	RUNX2	Coding	Exon 3	GCN	chr6:45390488	17	20 - 27	
Dentatorubral-pallidoluysian atrophy	DRPLA	AD	ATN1	Coding	Exon 5	CAG	chr12:7045880	7 - 34	49 - 88	
Early infantile epileptic encephalopathy 1	EIEE1	X	ARX	Coding	Exon 2	GCG	chrX:25031771	7 - 12	17 - 20	
Familial adult myoclonic epilepsy 1d	FAME1	AD	SAMD12	Intron	Intron 4/4	TTTCA or TTTGA	chr8:119379055	0.2 - 3 kb (Reference motif is TTTTA)	440 - 3680	Near AluSq2
Familial adult myoclonic epilepsy 3	FAME3	AD	MARCH6	Intron	Intron 1	TTTCA	5p15.2	12 (reference motif is TTTTA)	3 - 14 kb	
Familial adult myoclonic epilepsy 6	FAME6	AD	TNRC6A	Intron		TTTCA	chr16	0	29	Alu
Familial adult myoclonic epilepsy 7	FAME7	AD	RAPGEF2	Intron	Intron 14	TTTCA	chr4	0	Unknown	Alu
Fragile X tremor ataxia syndrome	FXTAS	X	FMR1	5' UTR	Exon 2	CGG	chrX:146993555	6 - 54	55 - 200	
Fragile-X site A	FRAXA	X	FMR1	5' UTR	Exon 2	CGG	chrX:146993555	6 - 54	200 - 1000+	

Fragile-X site E	FRAXE	X	FMR2	5' UTR	Exon 1	CCG	chrX:147582159	4-39	200 - 900	
Friedreich ataxia	FRDA	AR	FXN	Intron	Intron 1	GAA	chr9:71652201	6 - 32	200 - 1700	AluSx
Fuchs endothelial corneal dystrophy 3	FECD	AD	TCF4	Intron	Intron 1	CTG	chr18:53253385	10 - 40	50 - 150+	
Glutaminase deficiency	GD	AD	GLS	5'UTR	Exon 1	GCA	chr2:191745599	8 - 16	680 - 1500	
Hand-foot-uterus syndrome	HFG	AD	HOXA13	Coding	Exon 1	GCN	chr7:27239544	< 22		
Holoprosencephaly-5	HPE5	AD	ZIC2	Coding	Exon 3	GCN	chr13:100637703	< 25		
Huntington disease-like 2	HDL2	AD	JPH3	Coding	Exon 2	CTG	chr16:87637889	7 - 28	66 - 78	
Huntington's Disease	HD	AD	HTT	Coding	Exon 1	CAG	chr4:3076604	6 - 35	35+	
Kennedy Disease or Spinal-Bulbar Muscular Atrophy	SBMA	X	AR	Coding	Exon 1	CAG	chrX:66765159	9 - 35	38 - 62	
Mental retardation, FRA12A type	FRA12A	AD	DIP2B	5' UTR (Promoter)		CGG	12q13.1	6 - 23	> 350	
Machado Joseph Disease (Spinocerebellar Ataxia 3)	MJD	AD	ATXN3	Coding	Exon 9	CAG	chr14:92537355	13 - 36	61 - 84	
Mental retardation, X-linked	XLMR	XLR	SOX3	Coding	Exon 1	GCN	chrX:139586482	15	22 - 26	
Myoclonic epilepsy of Unverricht and Lundborg	EPM1	AR	CSTB	Promoter	Promoter	CCCCGCCCCGCG	chr21:45196324	2 - 3	40 - 80	
Myotonic dystrophy 1	DM1	AD	DMPK	3' UTR	Exon 3	CTG	chr19:46273463	5 - 37	50 - 10000	
Myotonic dystrophy 2	DM2	AD	ZNF9	Intron	Intron 1	CCTG	chr3:128891420	10 - 26	75 - 11000	Near AluSx
Neuronal intranuclear inclusion disease related disorders	NIIDRD	AD	NOTCH2NLC	5' UTR	Exon 1	GGC	chr1:145209324	< 40	66 - 517	
Oculopharyngeal muscular dystrophy	OPMD	AD	PABPN1	Coding	Exon 1	GCG	chr14:23790682	6 - 7	8 - 13	
Oculopharyngodistal myopathy	OPDM	AD	LRP12	5' UTR		CGG	chr8	13 - 45	Unknown	
Spinocerebellar Ataxia 1	SCA1	AD	ATXN1	Coding	Exon 8	CAG	chr6:16327865	6 - 38	39 - 82	
Spinocerebellar Ataxia 2	SCA2	AD	ATXN2	Coding	Exon 1	CAG	chr12:112036754	15 - 24	32 - 200	

Spinocerebellar Ataxia 6	SCA6	AD	CACNA1A	Coding	Exon 19	CAG	chr19:13318673	4 - 7	21 - 33	
Spinocerebellar Ataxia 7	SCA7	AD	ATXN7	Coding	Exon 4	CAG	chr3:63898361	4 - 35	37 - 306	
Spinocerebellar Ataxia 8	SCA8	AD	ATXN8	3' UTR	Exon 5	CTG	chr13:70713516	16 - 34	74+	
Spinocerebellar Ataxia 10	SCA10	AD	ATXN10	Intron	Intron 9/11	ATTCT	chr22:46191235	10 - 20	500 - 4500	Near AluSz
Spinocerebellar Ataxia 12	SCA12	AD	PPP2R2B	5' UTR	Exon 1	CAG	chr5:146258291	7 - 45	55 - 78	
Spinocerebellar Ataxia 17	SCA17	AD	TBP	Coding	Exon 3	CAG	chr6:170870995	25 - 42	47 - 63	
Spinocerebellar Ataxia 31	SCA31	AD	BEAN1	Intron	Intron 4/4	TGGAA	chr16:66524302	8 - 21 ((reference motif is AATAA, pathogenic repeat is an insert flanked by the reference motif)	2.5 - 3.8 kb	Near AluSx
Spinocerebellar Ataxia 36	SCA36	AD	NOP56	Intron	Intron 1	GGCCTG	chr20:2633379	3 - 8	1500 - 2500	
Spinocerebellar Ataxia 37	SCA37	AD	DAB1	Intron	Intron 3/16	ATTTC	chr1:57832716	< 30 (reference motif is ATTTT)	31 - 75	Near AluJb
Syndactyly	SD5	AD	HOXD13	Coding	Exon 1	GCN	chr2:176957787	< 22	>22	

Supplementary figure 1: Distributions of rare and common TRs in the 1000 Genomes dataset. (A) Percentage distribution of TRs into the rare and common subcategories. (B) Number of TRs per genome in each category. (C) Number of TRs as a function of sample size. (D) Frequency plot of the number of times a TR is observed in the cohort.

Supplementary figure 2: Distributions of TRs in different genomic regions; intergenic, intron, promoter, exon, and TTS in the 1000 Genomes dataset. (A) Percentage distribution of TRs into the genomic region subcategories. (B) Number of TRs per genome in each category. (C) Number of TRs as a function of sample size. (D) Frequency plot of the number of times a TR is observed in the cohort. (E) Odds ratios calculated by Fisher's exact test for TRs in different genomic regions, in both our dataset and the hg19 reference genome.

Supplementary figure 3: Distributions of TRs in *Alu* and non-*Alu* overlapping regions in the 1000 Genomes dataset. (A) Percentage distribution of TRs into the subcategories. (B) Number of TRs per genome in each category. (C) Number of TRs as a function of sample size. (D) Frequency plot of the number of times a TR is observed in the cohort. (E) Odds ratios calculated by Fisher's exact test for TRs in each category, in both our dataset and the reference genome.

Supplementary figure 4: Correlations between anchored IRR counts estimated by ExpansionHunter Denovo and number of repeats in the larger allele estimated by ExpansionHunter for a subset of samples with read counts above 5 at different disease loci (A) CANVAS motif AAAAG (B) CANVAS motif AAAGG (C) CANVAS motif AAGGG (D) SCA31 motif AAAAT (E) SCA37 motif AAAAT. Each correlation plot has an equation for the regression line and the spearman rho correlation coefficient.

Supplementary figure 5: Frequency of genomes with TRs of different motifs at the CANVAS locus.

Supplementary figure 6: (A) Anchored IRR counts in 1115 WGS samples, 1000 Genomes dataset, and Friedreich's ataxia positive control samples at the FXN gene intron 1 locus with repeat motif

GAA. Mann Whitney test p value and Cohen's d effect size are reported for each comparison. (B) ExpansionHunter version 3 estimated number of repeats for samples flagged as potentially pathogenic by EHDn. Dotted lines indicate the lowest reported number of repeats required for pathogenicity for each disease.

Supplementary figure 7: Functional categorization of genes with large TRs. (A) Subcellular localization of genes, and (B) molecular function of genes in the 1115 WGS samples, 1000 Genomes dataset, and hg19 reference. Pathways enriched in (C) 1115 WGS samples, (D) 1000 Genomes dataset, (E) hg19 reference, and (F) hg19 reference selected for repeats larger than 180bp.

Supplementary table 1: Depth-normalized anchored IRR counts of TRs genome wide in the 1,115 samples from the VAF cohort.

Supplementary table 2: Depth-normalized anchored IRR counts of TRs genome wide in the 2,504 samples from the 1000 Genomes cohort.

Supplementary table 3: Validation of ExpansionHunter Denovo results with PacBio long read sequence data. Ten TRs were randomly selected in 5 individuals from the 1000 Genomes dataset, and these were cross-referenced for validation in the PacBio long read data.

Supplementary table 4: Details regarding inheritance patterns, genomic positions, and ranges of repeats in healthy and disease individuals for the 44 known repeat expansion disorders.

Supplementary file 1: Bed file for use as an IGV custom annotation track to view TRs detected in the 1,115 samples from the VAF cohort.

Supplementary file 2: Bed file for use as an IGV custom annotation track to view TRs detected in the 2,504 samples from the 1000 Genomes cohort.
